# Targeting the programmed cell death 1: programmed cell death ligand 1 pathway reverses T cell exhaustion in patients with sepsis

**DOI:** 10.1186/cc13176

**Published:** 2014-01-04

**Authors:** Katherine Chang, Catherine Svabek, Cristina Vazquez-Guillamet, Bryan Sato, David Rasche, Strother Wilson, Paul Robbins, Nancy Ulbrandt, JoAnn Suzich, Jonathan Green, Andriani C Patera, Wade Blair, Subramaniam Krishnan, Richard Hotchkiss

**Affiliations:** 1 Departments of Anesthesiology, Washington University School of Medicine, 660 S. Euclid Ave, St Louis, MO 63110, USA; 2 Departments of Infectious Diseases and Vaccines, MedImmune LLC, Gaithersburg, MD 20878, USA; 3 Departments of Medicine, Washington University School of Medicine, 660 S. Euclid Ave, St Louis, MO 63110, USA; 4 Departments of Translational Medicine Oncology, MedImmune LLC, Gaithersburg, MD 20878, USA

## Abstract

**Introduction:**

A major pathophysiologic mechanism in sepsis is impaired host immunity which results in failure to eradicate invading pathogens and increased susceptibility to secondary infections. Although many immunosuppressive mechanisms exist, increased expression of the inhibitory receptor programmed cell death 1 (PD-1) and its ligand (PD-L1) are thought to play key roles. The newly recognized phenomenon of T cell exhaustion is mediated in part by PD-1 effects on T cells. This study tested the ability of anti-PD-1 and anti-PD-L1 antibodies to prevent apoptosis and improve lymphocyte function in septic patients.

**Methods:**

Blood was obtained from 43 septic and 15 non-septic critically-ill patients. Effects of anti-PD-1, anti-PD-L1, or isotype-control antibody on lymphocyte apoptosis and interferon gamma (IFN-γ) and interleukin-2 (IL-2) production were quantitated by flow cytometry.

**Results:**

Lymphocytes from septic patients produced decreased IFN-γ and IL-2 and had increased CD8 T cell expression of PD-1 and decreased PD-L1 expression compared to non-septic patients (*P*<0.05). Monocytes from septic patients had increased PD-L1 and decreased HLA-DR expression compared to non-septic patients (*P*<0.01). CD8 T cell expression of PD-1 increased over time in ICU as PD-L1, IFN-γ, and IL2 decreased. In addition, donors with the highest CD8 PD-1 expression together with the lowest CD8 PD-L1 expression also had lower levels of HLA-DR expression in monocytes, and an increased rate of secondary infections, suggestive of a more immune exhausted phenotype. Treatment of cells from septic patients with anti-PD-1 or anti-PD-L1 antibody decreased apoptosis and increased IFN-γ and IL-2 production in septic patients; (*P*<0.01). The percentage of CD4 T cells that were PD-1 positive correlated with the degree of cellular apoptosis (*P*<0.01).

**Conclusions:**

*In vitro* blockade of the PD-1:PD-L1 pathway decreases apoptosis and improves immune cell function in septic patients. The current results together with multiple positive studies of anti-PD-1 and anti-PD-L1 in animal models of bacterial and fungal infections and the relative safety profile of anti-PD-1/anti-PD-L1 in human oncology trials to date strongly support the initiation of clinical trials testing these antibodies in sepsis, a disorder with a high mortality.

## Introduction

Although most new therapeutic approaches to sepsis have focused on blocking the early hyper-inflammatory phase, recent studies have highlighted the profound immunosuppressive state that occurs after the initial stage of the disorder [1-4]. Numerous interacting mechanisms of immunosuppression occur in sepsis, including increased T regulatory cells, increased myeloid derived suppressor cells, apoptotic depletion of immune effector cells, and a shift from a TH1 to an anergic or TH2 immune phenotype [5-8]. Another recently recognized mechanism of immunosuppression in sepsis is T cell exhaustion [3]. T cell exhaustion was first described in states of chronic viral infection with persistent high levels of antigen exposure [9-11]. It is typified by the presence of T cells which have lost effector function, that is, they fail to proliferate, produce cytokines or induce cytotoxic cell death in targeted cells [10]. Exhausted T cells also have an increased tendency to undergo apoptosis because of changes in the ratio of pro- and anti-apoptotic Bcl-2 family members. One of the contributing factors for development of T cell exhaustion is signaling by the negative co-stimulatory molecule PD-1 (CD279), a member of the B7-CD28 super family, following interaction with its ligands PD-L1 (CD274) and PD-L2 (CD273) [9,11-13]. Following T cell activation, PD-1 is promptly induced and subsequently expressed on the surface of CD4 and CD8 T cells whereupon it interacts with PD-L1 and PD-L2. PD-L1 is broadly expressed on both hematopoietic and non-hematopoietic cells and its expression is significantly up-regulated during states of inflammation such as sepsis [11].

Although much of the focus and excitement of anti-PD-1 antibody therapy has been in the field of oncology, in which it has been demonstrated to be highly effective in inducing remissions in patients with a variety of malignancies [14,15], anti-PD-1 has also shown significant success in infectious disease. Multiple independent investigators have reported that blockade of the PD-1:PD-L1 pathway restores T cell effector function, increases IFN-γ production, prevents apoptosis and improves survival in various pathologic models of sepsis [16-20]. The present study compared and contrasted the ability of anti-PD-1 and anti-PD-L1 antibodies to decrease apoptosis and improve effector function in leukocytes from patients with sepsis. Another goal of the study was to determine if a correlation existed between lymphocyte apoptosis and putative mediators of apoptosis including lymphocyte PD-1 and PD-L1 expression and monocyte PD-L1 expression to gain insight into possible mechanisms for apoptotic cell death and the lymphocytopenia that typically accompany sepsis.

## Methods

### Patient selection

#### Septic patients

Patients at Barnes Jewish Hospital who were older than 18 years of age and who fulfilled a consensus panel definition of sepsis [21] were included in the study (Table 1). Sepsis was defined as the presence of systemic inflammatory response syndrome (SIRS) and a known or suspected source of infection. Patients with HIV infection, viral hepatitis, or who were receiving immunosuppressive medications (except corticosteroids at a dose of <10 mg prednisone or equivalent per day) were excluded. Consent for blood draws was obtained from the patient or a legally authorized representative.

**Table 1:** Patient characteristics

		**Septic**	**Non-septic**
**# Patient**		43	15
**Age**			
	Median	64	53
	IQR	53 to 71	42 to 77
**Gender**			
	Male	21	9
	Female	22	6
**APACHE II**			
	Median	16	7
	IQR	13 to 211	6 to 9
**SOFA (Sequential Organ Failure Assessment)**			
	Median	8	3
	IQR	5 to 10	2 to 4
**ALC (Absolute lymphocyte count)** (cells × 10^3^/microliter)			
	Median	1.0	0.9
	IQR	0.7 to 1.4	0.7 to 1.1
**INR (International normalized ratio)** (seconds)			
	Median	1.32	1.19
	IQR	1.18 to 1.40	1.13 to 1.33
**Serum creatinine** mg/dl			
	Median	0.96	0.7
	IQR	0.74 to 1.65	0.6 to 1.0
**Length of ICU stay**			
	Median	10	5
	IQR	6 to 16	2 to 16
**Mortality (%)**			
	Survived	34 (79)	13 (87)
	Expired	9 (21)	2 (13)
**Vasopressor - dependent shock**		29	
**Admission ICU diagnosis**			
	Community-acquired pneumonia	14	
	Ventilator-associated pneumonia	6	
	Peritonitis	15	
	Wound infection	2	
	Line infection	6	
	Trauma		10
	Post-op (major surgery)		3
	Intra-cranial hemorrhage		2
**Co-morbidities**			
	Diabetes	17	5
	Heart disease	35	14
	Morbid obesity	2	0
	Neurologic	6	3
	Renal disease	6	0
	Respiratory	14	2
	Liver	1	0

*ALC,* absolute lymphocyte count; *APACHE* II*,* Acute Physiology and Chronic Health Evaluation II; *INR,* International Normalized Ratio; *IQR,* Interquartile range; *SOFA*, Sequential Organ Failure Assessment.

#### Critically-ill non-septic patients

Control subjects consisted of critically-ill non-septic patients admitted to the ICU for care following major surgery, trauma or myocardial ischemia (Table 1). Exclusion criteria were identical to that for patients with sepsis. Consent for blood draws was obtained from the patient or a legally authorized representative.

All protocols were approved by the Washington University Institutional Review Board.

### Blood collection and processing

Patients provided consent for a maximum of four blood samples (5 ml/sample) obtained serially at days 1 to 3 after admission to the ICU (‘A’), days 4 to 7 (second blood draw, ‘B’), days 8 to 12 (third blood draw, ‘C’), and days 13 to 21 (fourth blood draw, ‘D’) after sepsis onset. The same serial blood draw protocol was used in non-septic patients. Heparinized blood was collected through an indwelling central venous or arterial catheter or by peripheral venipuncture. The blood was immediately transported and processed in the laboratory. Peripheral blood mononuclear cells (PBMCs) were isolated by density gradient separation. Plasma was collected and stored at -80°C for subsequent analysis. The cells were washed and resuspended in RPMI 1640 and processed for immunostaining or overnight incubation as previously described.

### Flow cytometry

Antibodies for flow cytometric determinations were purchased from BioLegend (San Diego, CA, USA), BD Biosciences (San Diego, CA, USA) or eBiosciences (San Diego, CA, USA). Cellular expression of PD-1 and PD-L1 on acutely isolated PBMCs was performed on the day of blood draw. Lymphocytes were identified by forward scatter (FSC) and side scatter (SSC) properties as described previously [3]. Monocytes were identified by FSC and SSC properties and by CD14+ immunostaining. T cell subsets were further identified by CD3+, CD4+ or CD8+ immunostaining. NK cells were identified as CD3-/CD56+ while natural killer T (NKT) cells were identified as CD3+/CD56 + .

### Effects of anti-PD-1 and anti-PD-L1 on lymphocyte apoptosis

A total of approximately 1 × 10^7^ cells were incubated overnight. Cells were treated with either isotype-control antibody, anti-PD-1 antibody or anti-PD-L1 antibody. Anti-PD-1 antibody and anti-PD-L1 antibody were provided by MedImmune and were all human IgG1. The effect of anti-PD-1 and anti-PD-L1 antibody on lymphocyte apoptosis following overnight incubation was quantitated via the TUNEL assay as previously described [18].

### Effects of anti-PD-1 and anti-PD-L1 on stimulated IFN-γ and IL-2 production

PBMCs that had undergone overnight incubation with either isotype-control antibody, anti-PD-1 antibody or anti-PD-L1 antibody were stimulated with PMA/ionomycin plus brefeldin for 5 h as previously described [22,23]. Following stimulation, cells were washed, stained with anti-CD3 and anti-CD56 antibodies, fixed with 1% paraformaldehyde, permeabilized with 1X perm/wash (BioLegend) and stained with fluorescently labeled anti-IFN-γ or anti-IL-2 antibodies.

### Patient hematologic values

Depending upon severity of illness, ICU patients have daily complete blood count analysis performed as part of the standard of care. Patient clinical laboratory values that were recorded in this study included absolute lymphocyte, absolute monocyte, and absolute granulocyte cell counts and were quantitated in the clinical laboratories at Barnes Jewish Hospital (see Additional file 1: Table S1).

### Definition of hospital-acquired secondary infections

#### Secondary infections

Data on nosocomial infections occurring while patients were in the ICU were abstracted from medical records using standard Center for Disease Control case definitions (http://www.cdc.gov/hai/). Identification of secondary infections was performed by an individual who was blinded to patient stratification.

### Consent

Written informed consent was obtained from the patient or, if the patient was unable to provide consent, their relative for publication of their individual details and accompanying images in this manuscript. The consent form is in the patients’ clinical notes and a copy is also held by the authors and is available for review by the Editor-in-Chief.

### Statistical analysis

Data were analyzed with the statistical software Prism (GraphPad, San Diego, CA, USA). Data are reported as the mean ± SEM. For comparison of two groups, the Student’s *t*-test was employed. A paired *t*-test was used when comparing samples from the same patient which were treated identically except for incubation with either anti-PD-1 or anti-PD-L1 antibodies. One-way ANOVA with Tukey’s multiple comparison tests was used to analyze data in which there were more than two groups. Significance was reported at *P* <0.05.

## Results

### Patient demographics

Relevant clinical and laboratory values for septic and critically-ill non-septic patients regarding median age, gender, sites of infection, severity of illness scores, mortality, length of ICU stay and so on are provided in Table 1. Additional patient data are presented in Additional file 1: Table S1 and Additional file 2: Table S2. A total of 43 septic patients were included in the study. Thirty-nine of the 43 septic patients were located in ICUs; 3 septic patients were located in lesser acuity treatment areas including observation units. Fifteen critically-ill non-septic patients were included in the study. Two non-septic critically-ill patients became septic with ventilator-associated pneumonia during their initial ICU admission. Data from these two patients are included in both septic and non-septic columns based upon their particular phase of illness, that is, non-septic or septic phase. Most non-septic patients did not remain in the ICU past four days and therefore, only one blood draw was obtained in these patients. Mortality in the septic and critically-ill non-septic patients was 21% and 13%, respectively (Table 1). The most common causes of sepsis were community acquired pneumonia and peritonitis (Table 1).

### Sepsis increased CD8 PD-1 and monocyte PD-L1 expression

**Figure 1:**
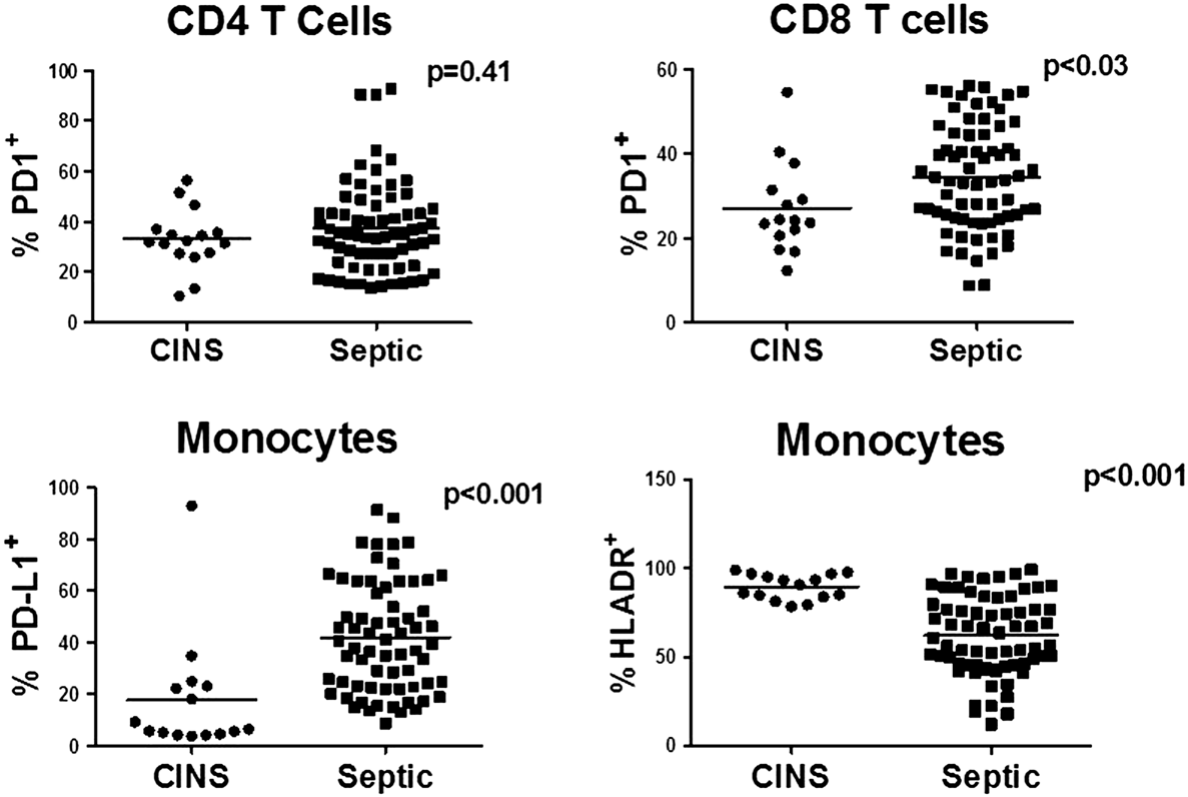
**PD-1, PD-L1 and HLA-DR expression in septic and non-septic patients.** Septic and non-septic patients were identified and heparinized blood samples obtained at a maximum of four time points during their septic course. Peripheral blood mononuclear cells were stained for lymphocyte (CD4, CD8) and monocyte markers (CD14). Immunostaining was also performed for programmed cell death 1 (PD-1), programmed cell death ligand 1 (PD-L1) and human leukocyte antigen-DR (HLA-DR). Flow cytometry revealed an increase in PD-1 and PD-L1 expression in CD8 T cells and monocytes from septic versus non-septic patients. HLA-DR expression was decreased in monocytes from septic versus non-septic patients as well. Data are from 43 septic (70 data points) and 16 non-septic patients (16 data points). Septic and non-septic patients had up to four serial blood samples obtained depending upon the duration of their illness and/or discharge from the ICU; - first draw = days 1 to 3 after admission to the ICU, days 4 to 7 (second blood draw), days 8 to 12 (third blood draw), and days 13 to 21 (fourth blood draw) after sepsis onset. Most non-septic patients were discharged from the ICU within four to five days and, therefore, they had less serial blood samples obtained compared to septic patients.

**Figure 2:**
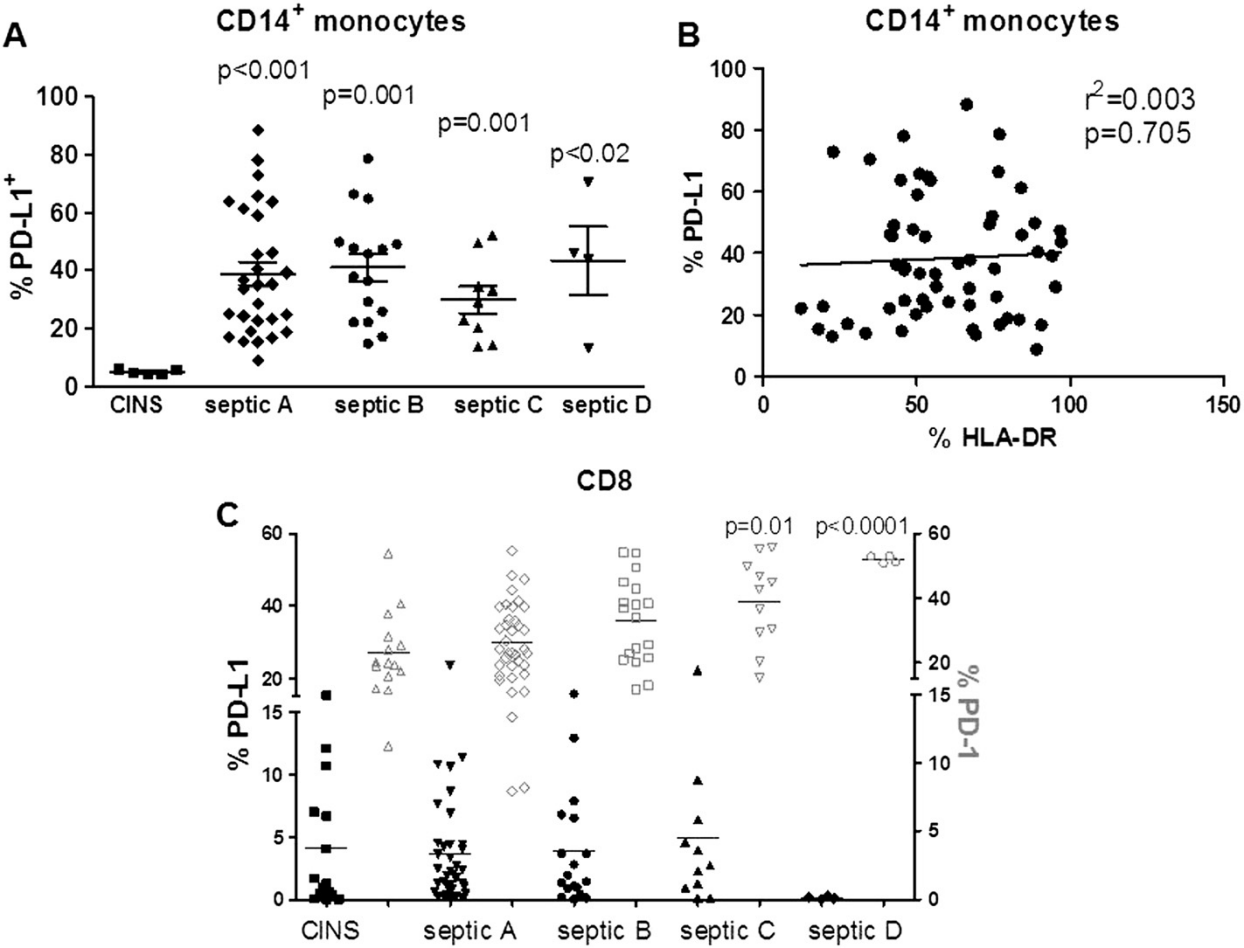
**Markers of immune exhaustion increase with protracted sepsis.** Flow cytometry revealed an increase in expression of immune exhaustion markers over time in the ICU. **A)** Monocyte PD-L1 expression was increased in septic patients compared to critically-ill non-septic patients (CINS) patients. In contrast to changes in programmed cell death 1 (PD-1) and programmed cell death ligand 1 (PD-L1) expression on CD8 T cells during sepsis, there was no change in the expression of PD-L1 during the septic time period. **B)** Comparison of monocyte PD-L1 expression and human leukocyte antigen-DR (HLA-DR) expression did not show any correlation in patients with sepsis. **(C)** PD-1 expression increased as PD-L1 expression decreased on CD8 T cells in samples from septic patients over the course of their sepsis, that is, time points A (days 1 to 3), B (days 4 to 7), C (days 8 to 12) and D (days 13 to 21). CD8 T cell PD-1 expression was higher in septic versus critically-ill non-septic patients (CINS). CD8 T cell expression of PD-L1 fell to very low levels at time point D in septic patients compared to CINS patients and at time points A-C in septic patients. *P*-values in 2A and 2C are comparison of septic samples with CINS for each draw.

Examination of PD-1 expression on CD4 and CD8 T cells showed that sepsis caused an increase in CD8 but not CD4 PD-1 expression compared to non-septic patients (Figure 1, Additional file 3: Figure S1). The percentage of monocytes that were expressing PD-L1 was increased over two-fold in septic versus non-septic patients (Figure 1). As is characteristically described in patients with sepsis [4], monocyte HLA-DR expression was significantly decreased in septic versus non-septic patients, *P* <0.001, (Figure 1). Monocyte HLA-DR expression remained depressed throughout the duration of sepsis (Additional file 4: Figure S2). Similarly, PD-L1 on monocytes from septic patients remained elevated but did not change over time (Figure 2A), and there was no correlation between monocyte HLA-DR expression and PD-L1 expression (Figure 2B). Analysis of PD-1 and PD-L1 expression over time in septic patients revealed that expression of PD-1 on CD8+ T cells increased as PD-L1 decreased during their stay in ICU (Figure 2C). Furthermore, the subset of septic patients with higher PD-1 together with lower PD-L1 expression on CD8 cells (‘CD8+ PD-1^high^ PD-L1^low^’) in which CD8+ PD-1 was ≥36% and CD8+ PD-L1was ≤5% expression also had reduced levels of HLA-DR expression on CD14+ monocytes compared with CD8+ PD-1^low^ PD-L1^high^ septic patients (defined as CD8+ PD-1 ≤36% and CD8+ PD-L1 ≥5% expression) and critically ill non-septic patients (Additional file 5: Figure S3A). These values for CD8+ PD-1^high^ PD-L1^low^ were chosen based upon the mean values for CD8+ PD-1 and CD8+ PDL1 expression for critically-ill non-septic patients. In other words, CD8+ PD-1 ≥36% and CD8+ PD-L1 ≤5% represented the values for septic patients which were above the mean values for CD8+ PD-1 expression and below the mean CD8+ PD-L1 expression for critically-ill non-septic patients. This association of CD8+ PD-1 and PD-L1 expression with HLA-DR expression was further validated by reciprocal analysis of HLA-DR expression on CD14 monocytes. In this setting, monocytes from septic patients which had a <50% expression of HLA-DR had a higher proportion of CD8+ PD-1^high^ PD-L1^low^ T cells than septic patients with greater than 50% HLA-DR + monocytes (data not shown). CD8+ PD-1^high^ PD-L1^low^ patient samples were detected at all time points tested during their septic condition, but increased over time (26.7%, 37.5%, 40% and 100% of samples per blood draw A to D respectively). Interestingly, this same subgroup of septic patients had an increased rate of secondary infections, that is, ventilator associated pneumonia (VAP) and peritonitis compared with other septic patients (*P* <0.05, Additional file 5: Figure S3B). These data support the idea that septic patients are a heterogeneous population at different stages of disease and with differing immunologic status upon presentation to the ICU, but develop progressively increasing levels of immune exhaustion with protracted sepsis.

### Anti-PD-1 and anti-PD-L1 decreased sepsis-induced apoptosis in lymphocytes

**Figure 3:**
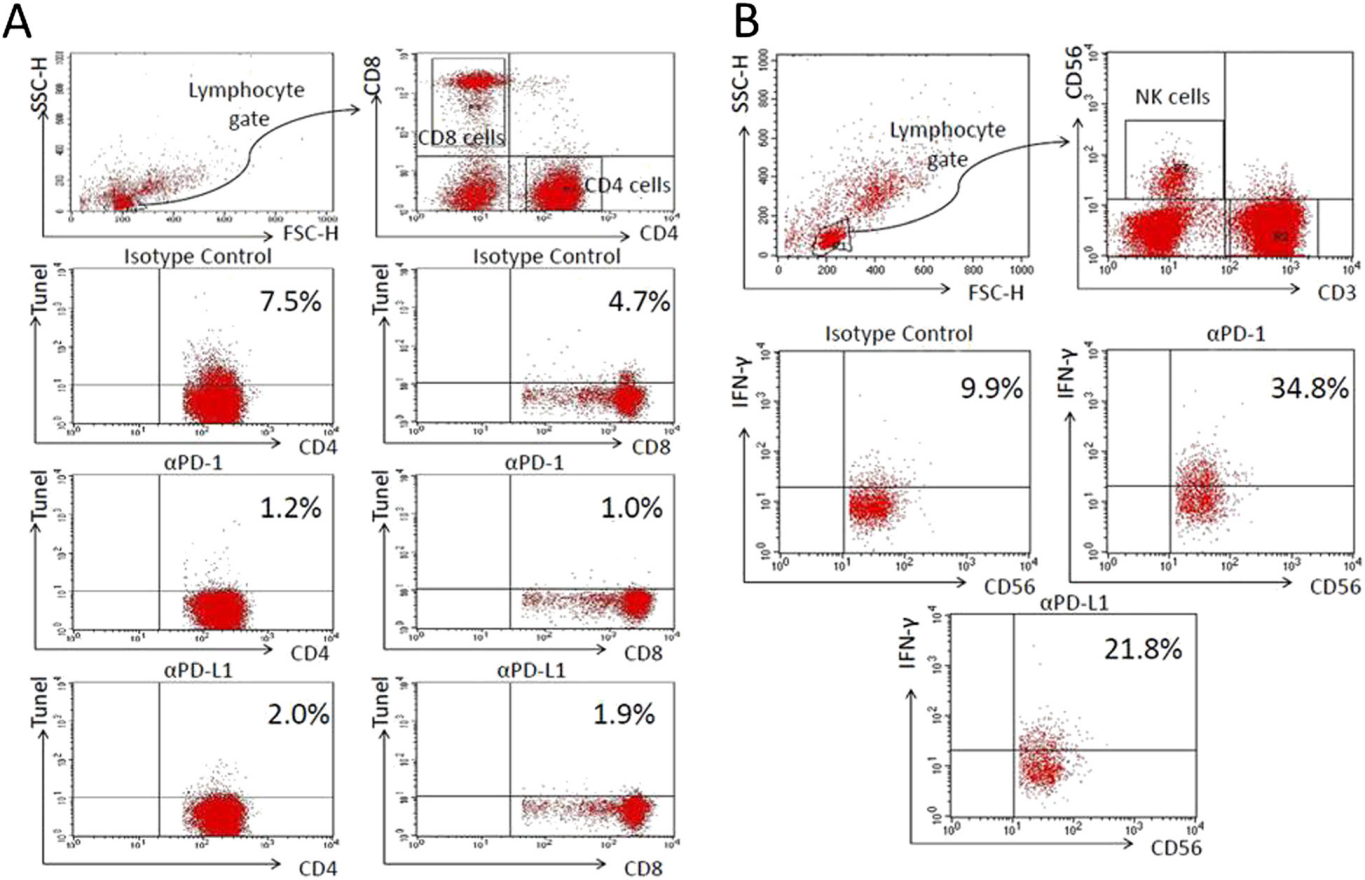
**Representative flow histograms of lymphocyte gating strategy and detection of apoptosis and IFN-γ.** Peripheral blood mononuclear cells (PBMCs) from a septic patient were plated overnight with isotype control antibody, anti-programmed cell death 1 (PD-1) or anti-programmed cell death ligand 1 (PD-L1) antibody. **A**: Cells were immunostained for CD4 and CD8 T cells and TUNEL assay performed. The lymphocyte fraction in the PBMCs was identified by characteristic forward and side scatter properties and CD4 and CD8 T cells identified by cell-specific antibodies. Apoptosis in CD4 T cells incubated in inactive isotype control antibody was 7.5%. Anti-PD-1 and anti-PD-L1 decreased CD4 apoptosis to 1.25 and 2.0%, respectively. A similar protective effect was seen in CD8 T cells. **B**: PBMCs from a septic patient were incubated overnight with isotype control antibody, anti-PD-1 or anti-PD-L1 antibody. The following morning, cells were stimulated with PMA/ionomycin plus brefeldin for 5 h, washed, immunostained with phenotypic markers to CD3 and CD56, fixed and stained for intracellular interferon (IFN)-γ. The lymphocyte gate was identified by characteristic forward and side scatter properties. Natural killer (NK) cells were identified as CD3 negative CD56 positive. The percentage of NK cells that were positive for IFN-γ that were incubated in inactive isotype control antibody was 9.9%. Treatment with anti-PD-1 and anti-PD-L1 increased the percentage of NK cells that were IFN-γ positive to 34.8 and 21.8%, respectively.

**Figure 4:**
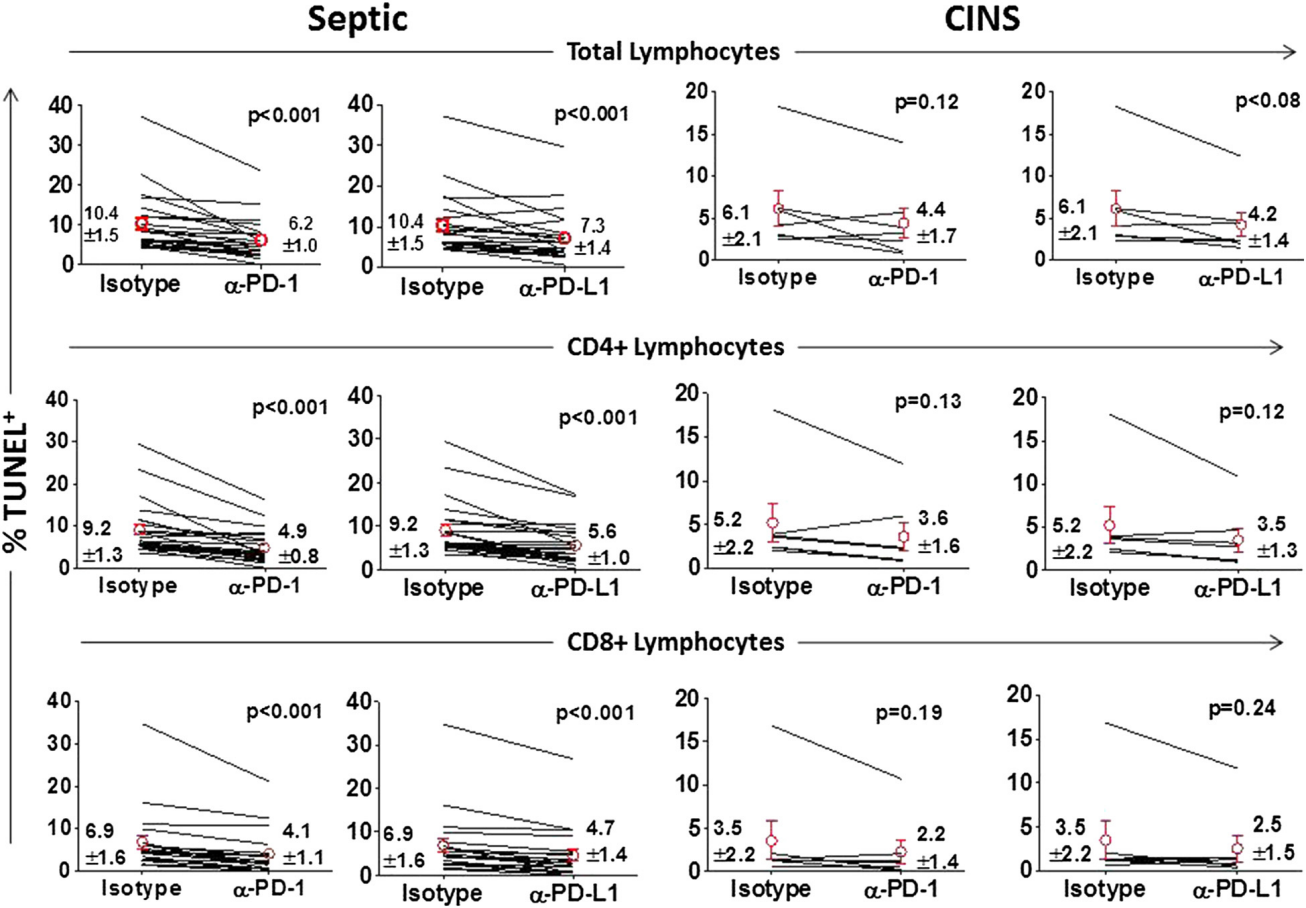
**Anti-PD-1 and anti-PD-L1 decrease lymphocyte apoptosis in sepsis.** Peripheral blood mononuclear cells (PBMCs) from septic or critically-ill non-septic (CINS) patients were incubated overnight in media containing isotype control antibody, anti-programmed cell death 1 (−PD-1) or anti-programmed cell death ligand 1 (−PD-L1) antibody. The following morning, cells were washed and underwent immunostaining followed by detection of apoptosis via TUNEL assay. Compared to treatment with inactive isotype control antibody, incubation with anti-PD-1 or anti-PD-L1 antibody decreased apoptosis in total lymphocytes, CD4 and CD8 T cells from septic patients; *P* <0.001. There was no significant effect of anti-PD-1 or anti-PD-L1 in CINS patients, possibly due to the fact that baseline apoptosis in the non-septic patients was so low. Values shown are the mean ± SEM values for all time points for 19 septic and 7 CINS patients. Circles indicate mean per group.

Apoptosis was quantitated in patient lymphocytes after overnight incubation with isotype control antibody, anti-PD-1 antibody or anti-PD-L1 antibody. Quantitation of apoptosis in total lymphocytes, that is, all lymphocytes present in the lymphocyte gate identified by forward and side scatter on flow cytometry (Figure 3A) and consisting primarily of CD4+, CD8+, NKT cells and NK cells was examined. Total lymphocyte apoptosis was increased by approximately 70% in septic patients when compared to non-septic patients after overnight incubation in isotype (inactive) control antibody, that is, 10.4 ± 1.5% in septic patients versus 6.1 ± 2.1% in non-septic patients (*P* <0.01), (Figure 4, Additional file 6: Figure S4). Compared to lymphocytes incubated with isotype control antibody, lymphocytes incubated in media containing anti-PD-1 or anti-PD-L1 antibody had a highly significant decrease in apoptosis, *P* <0.001, (Figure 4). No effect of anti-PD-1 or anti-PD-L1 antibody on lymphocyte apoptosis was seen in samples from non-septic patients, possibly due to their lower level of baseline apoptosis which was often less than 5%, (Figure 4). A highly similar effect of anti-PD-1 and anti-PD-L1 antibody on sepsis-induced apoptosis was observed in CD4 and CD8 T cells from septic patients (Figure 4).

### Anti-PD-1/anti-PD-L1 ameliorate sepsis-induced impairment in production of IFN-γ and IL-2

**Figure 5:**
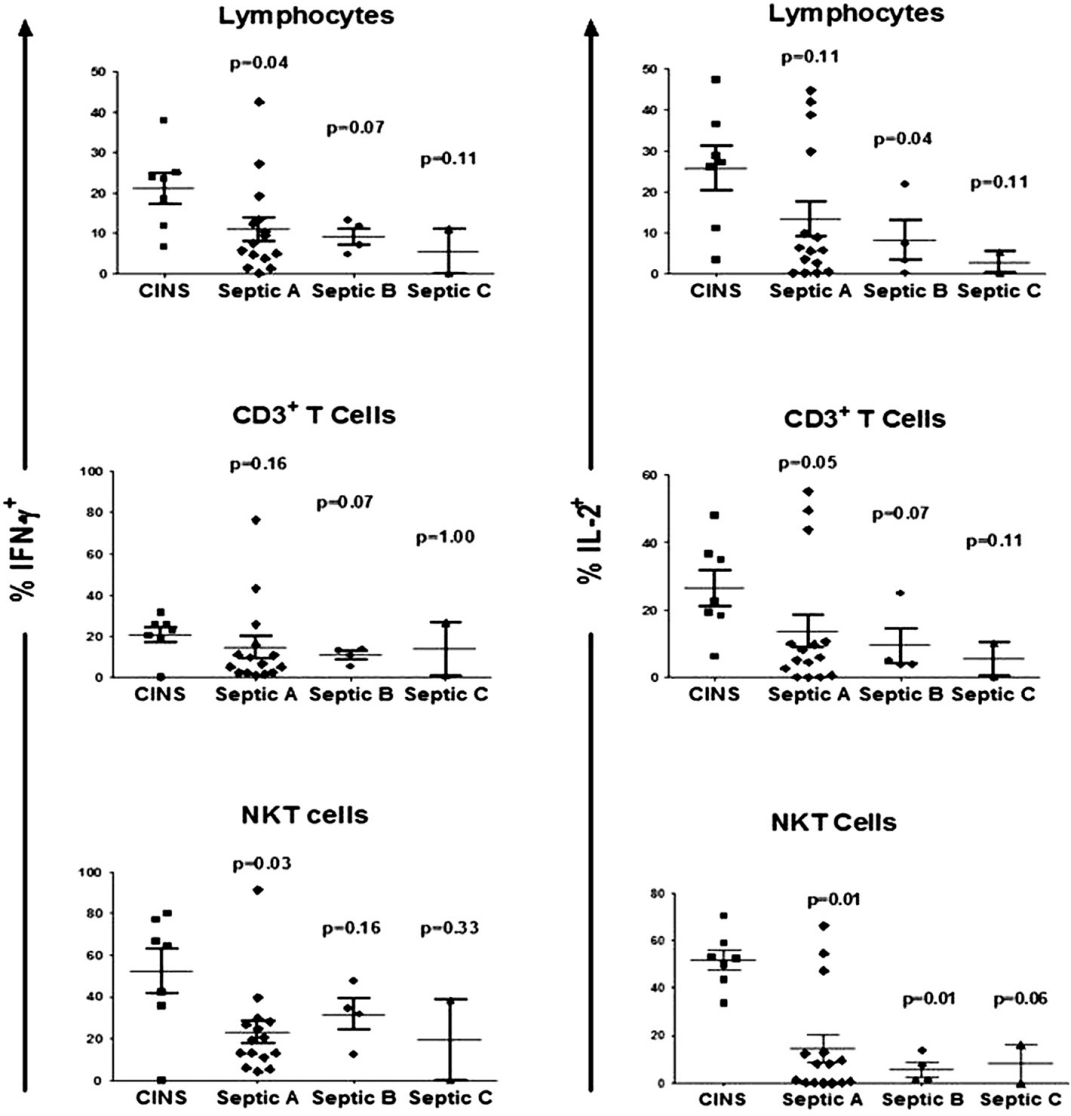
**Sepsis impairs lymphocyte IFN-γ and IL-2 production.** Peripheral blood mononuclear cells (PBMCs) from septic or critically-ill non-septic (CINS) patients were incubated overnight in media containing isotype control antibody. Blood from septic patients was obtained at three time points during the sepsis, that is, days 1 to 3 (Septic A), days 4 to 7 (Septic B) and days 8 to 12 (Septic C). Samples from days 13 to 21 (Septic D) were not tested. The following morning, cells were stimulated with PMA/ionomycin plus brefeldin for 5 h, washed, immunostained with phenotypic markers to CD3 and CD56, fixed and stained for intracellular interferon (IFN)-γ or interleukin (IL)-2. Flow cytometric analysis revealed a persistent decrease in the percentage of total lymphocytes and natural killer T (NKT) cells that were IFN-γ positive in septic compared to CINS patients throughout most of the septic duration. The difference in IFN-γ production in septic versus CINS patients did not quite reach statistical significance for CD3 T cells, *P* = 0.07. A similar pattern of decreased IL-2 production in septic versus CINS patients occurred at all septic time points. Data are from 15 septic (21 data points) and 7 CINS patients (7 data points) obtained during their illness. *P*-values shown are comparison of septic samples with CINS for each draw.

**Figure 6:**
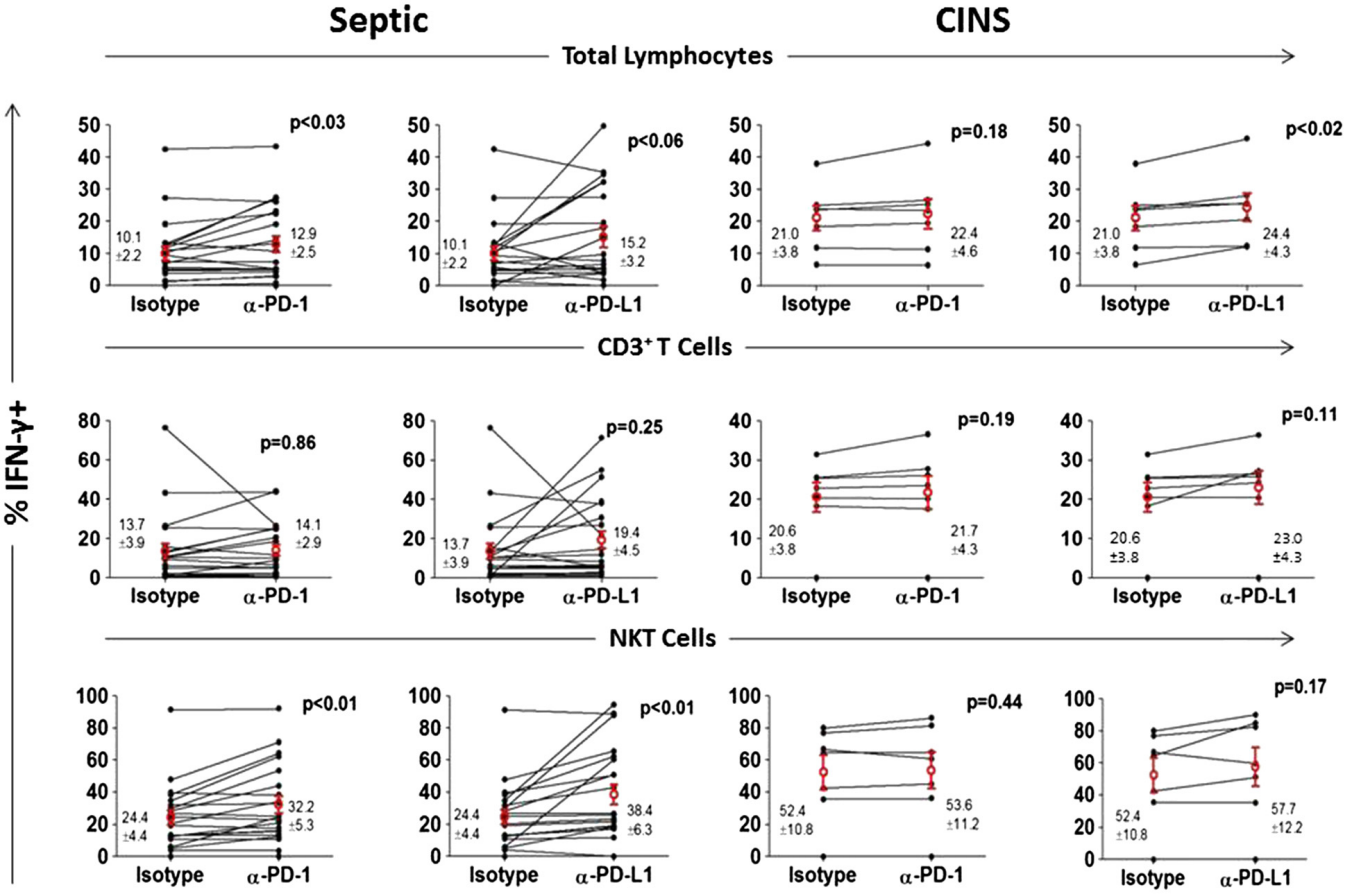
**Anti-PD-1 and anti-PD-L1 antibodies increase IFN-γ production in sepsis.** Peripheral blood mononuclear cells (PBMCs) from septic or critically-ill non-septic (CINS) patients were incubated overnight in media containing isotype control antibody, anti- programmed cell death 1 (−PD-1) or anti- programmed cell death ligand 1 (−PD-L1) antibody. The following morning, cells were stimulated with PMA/ionomycin plus brefeldin for 5 h, washed, immunostained with phenotypic markers to CD3 and CD56, fixed and stained for intracellular interferon (IFN)-γ. Flow cytometric analysis revealed that, compared to inactive isotype control antibody, both anti-PD-1 and anti-PD-L1 antibody caused an increase in the percentage of total lymphocytes and natural killer T (NKT) cells that were IFN-γ positive. Anti-PD-1 had no significant effect in CINS patients while anti-PD-L1 increased IFN-γ in total lymphocytes only. Note that IFN-γ production was higher in CINS patients compared to septic patients when incubated with inactive isotype control antibody. Data are from 15 septic patients (21 data points) throughout their illness - all blood draws. Values shown are mean ± SEM values for all time points. Circles indicate mean per group.

**Figure 7:**
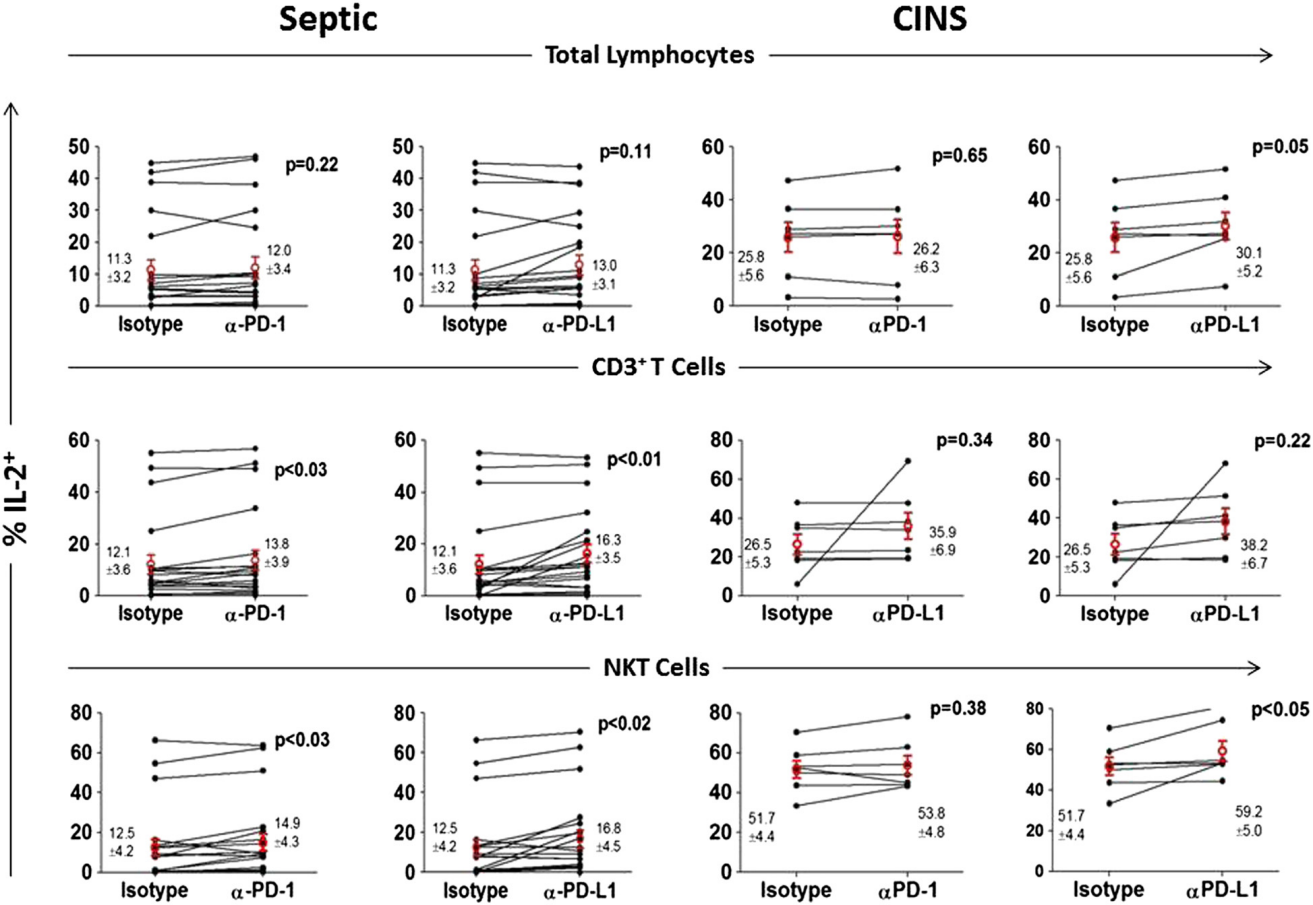
**Anti-PD-1 and anti-PD-L1 antibodies increase IL-2 production in sepsis.** Peripheral blood mononuclear cells (PBMCs) from septic or critically-ill non-septic (CINS) patients were incubated overnight in media containing isotype control antibody, anti- programmed cell death 1 (PD-1) or anti- programmed cell death ligand 1 (PD-L1) antibody. The following morning, cells were stimulated with PMA/ionomycin plus brefeldin for 5 h, washed, immunostained with phenotypic markers to CD3 and CD56, fixed and stained for intracellular interleukin (IL)-2. Flow cytometric analysis revealed that, compared to inactive isotype control antibody, both anti-PD-1 and anti-PD-L1 antibody caused an increase in the percentage of CD3 and natural killer T (NKT) cells that were IL-2 positive. Anti-PD-L1 had an effect to increase IL-2 production in total lymphocytes and NKT cells in CINS patients. Note that IL-2 production was higher in CINS patients compared to septic patients when incubated with inactive isotype control antibody. Data are from 15 septic patients (21 data points) throughout their illness. Values shown are the mean ± SEM values for all time points. Circles indicate mean per group.

PBMCs from septic or non-septic patients were divided equally into wells and incubated overnight with isotype control antibody, anti-PD-1 antibody or anti-PD-L1 antibody. The next morning, cells were washed, stained for various lymphocyte subsets and stimulated (see Methods). Compared to non-septic patients, septic patients tended to have persistently decreased intracellular production of IFN-γ and IL-2 at multiple time points during the course of their sepsis (Figures 3B and 5). This defect occurred in total lymphocytes, CD3 lymphocytes and NKT cells. Overnight incubation of PBMCs from septic patients showed a significant effect of anti-PD-1 and anti-PD-L1 antibodies to increase IFN-γ production in total lymphocytes and NKT cells compared to cells incubated with isotype control antibody (Figure 6). Examination showed that a subset of patients’ samples responded to anti-PD-1 or anti-PD-L1. Anti-PD-1 and anti-PD-L1 had similar effects to increase IL-2 production in specific lymphocyte subsets (Figure 7). Anti-PD-1 and anti-PD-L1 had minimal effect on IFN-γ or IL-2 production in lymphocytes from non-septic patients (Figures 6 and 7).

### Correlation of apoptosis, absolute lymphocyte count and PD-1 expression

**Figure 8:**
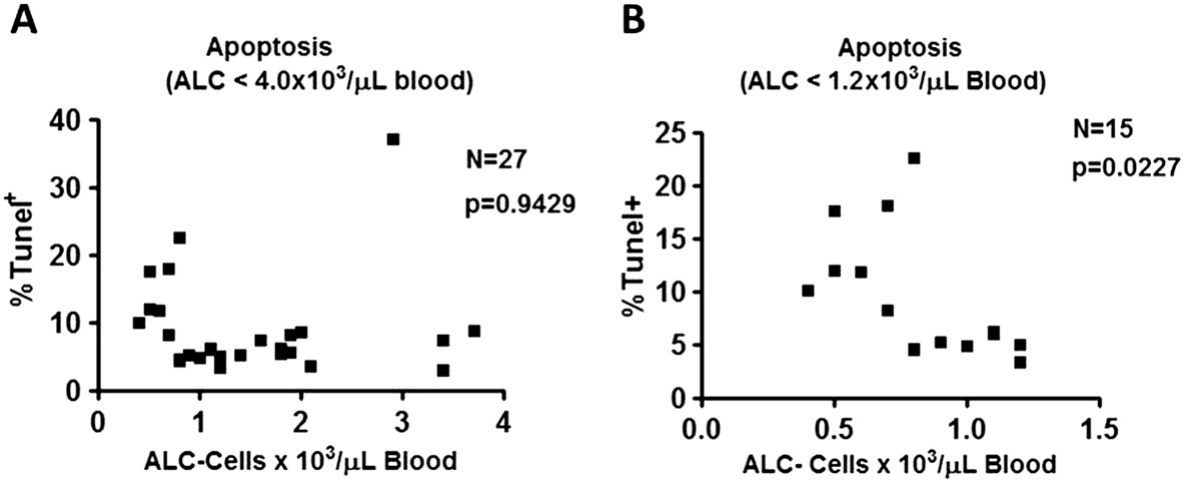
**Correlation of absolute lymphocyte count and apoptosis.** A low absolute lymphocyte count (ALC) is a frequent finding in sepsis and correlates with outcome (see Discussion). We examined the relationship between ALC and apoptosis as determined by the Tunel assay. There was no relationship of apoptosis (percent Tunel positive cells) and the ALC in septic patient considered as a whole **(A)**. If the correlation of apoptosis and ALC was restricted to septic patients who had an ALC below the lower limit of normal at Barnes Jewish Hospital (1.2 × 10^3^ lymphocytes/μL blood), there was a correlation such that patients with the lowest ALCs had the highest percent of Tunel + (apoptotic) lymphocytes. **(B)** There was a statistical correction between ALC and lymphocyte apoptosis in septic patients who had an ALC less than 1.2 × 10^3^ cells/μL blood (the lower limit of normal at our hospital).

**Figure 9:**
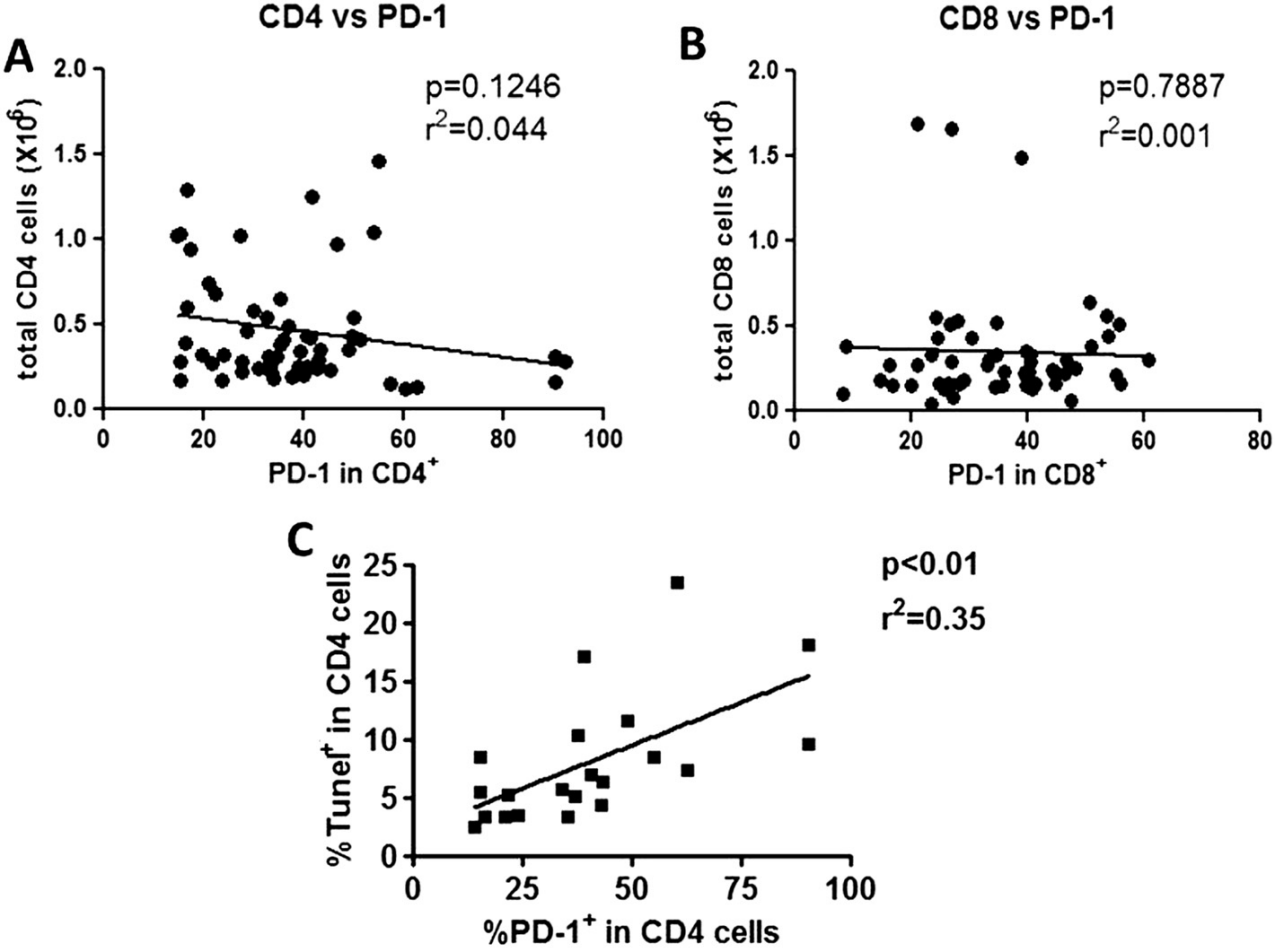
**Correlation of PD-1 expression and absolute cell counts and apoptosis. (A and B)**: Since anti-programmed cell death 1 (PD-1) antibody decreased lymphocyte apoptosis (Figure 4), we investigated the correlation between PD-1 expression on CD4 and CD8 T cells and absolute CD4 and CD8 T cells, respectively. Neither CD4 nor CD8 PD-1 expression correlated with absolute (total) CD4 or CD8 T cell counts respectively. **(C)**: We also investigated the correlation between PD-1 expression and apoptosis by examining the relationship between percent PD-1 + CD4 T cells and the percent Tunel + CD4 T cells. At the time of the first blood draw in septic patients (days 1 to 3), there was a positive correlation between PD-1 expression on CD4 T cells and the percent of apoptotic cells (Tunel+).

A characteristic hematologic finding in patients with sepsis is an apoptosis-induced reduction in their absolute lymphocyte count (ALC), often to values that are less than 20 to 30% of that for healthy controls [5,24,25]. Importantly, persistent lymphopenia in sepsis is associated with increased mortality, and lymphocyte depletion (as reflected by lymphopenia) may contribute to morbidity and mortality by impairing host immunity [26,27]. There are multiple mechanisms for the low ALC in sepsis, including recruitment of lymphocytes to sites of infection and apoptosis. We correlated lymphocyte apoptosis with the ALC (Figure 8). Although there was no correlation between ALC and lymphocyte apoptosis when samples from all septic patients as a group were included (Figure 8A), if the study population was restricted to patients who had an ALC less than 1.2 × 10^3^ cells/μL blood (the lower limit of normal for ALC at our medical center), there was a statistical correlation between low ALC and the percent of lymphocytes undergoing apoptosis (Figure 8B). In other words, septic patients with low ALC tend to have increased rates of lymphocyte apoptosis. In order to examine the potential role for PD-1:PD-L1interaction in lymphocyte apoptosis, one possible cause of lymphopenia in sepsis, we investigated the correlation between PD-1 expression on CD4 and CD8 T cells and absolute numbers of CD 4 and CD8 T cells. There was no correlation between CD4 or CD8 PD-1 expression and total numbers of CD4 or CD8 T cells or ALC (Figure 9A, B and Additional file 7: Figure S5). Finally, we examined the correlation of percent PD-1+ CD4 or CD8 T cells and apoptosis as reflected by the degree of Tunel positivity. At the first blood draw in septic patients, that is, days 1 to 3 after ICU admission (time point A), when lymphocyte apoptosis was maximum (Additional file 6: Figure S4), there was a correlation between the percent Tunel + CD4 T cells and the expression of PD-1 on CD4 T cells (Figure 9C). This correlation did not exist for CD4 or CD8 T cells at other time points (Additional file 8: Figure S6).

## Discussion

The present results show that blockade of either PD-1 or its ligand PD-L1 reverses two pathophysiologic hallmarks of sepsis. Anti-PD-1 and anti-PD-L1 antibodies markedly decreased sepsis-induce lymphocyte apoptosis and restored the ability of immune effector cells to produce cytokines that are essential for host immunity. These *in vitro* findings in patient leukocytes strengthen the concept that blockade of the PD-1:PD-L1 pathway offers a promising new approach in the treatment of sepsis [17,28]. Although most previous therapeutic trials in sepsis have focused on blockade of the initial hyper-inflammatory phase, there is increased recognition that if patients survive this initial stage of the disorder, they progress to an immunosuppressive state [4,28-32]. New treatment protocols have resulted in the fact that the majority of deaths in sepsis now occur after the first four days of sepsis (the hyper-inflammatory phase) and during the immunosuppressive phase [33]. Furthermore, microbiologic studies of patients dying of sepsis showed that over 50% of the infecting organisms were classified as opportunistic pathogens (opportunistic bacteria and fungi), a finding which is highly compatible with impaired immunity [33]. In this setting, use of immuno-adjuvant agents including anti-PD-1 or anti-PD-L1 antibodies is a logical approach to restore host immunity and potentially improve survival.

Research into the mechanistic basis of immunosuppression in sepsis has determined that multiple overlapping etiologies exist including increased T regulatory and myeloid derived suppressor cells and apoptotic depletion of T and B cells [5-8]. A relatively newly recognized etiology of immunosuppression in sepsis is T cell exhaustion. T cell exhaustion was first reported in animal models of chronic viral infection and was thought to be due to persistent exposure to high levels of antigen [9-11]. Patients with sepsis often have a protracted course with primary and secondary infections, a scenario that likely includes persistent high circulating antigens thereby facilitating development of T cell exhaustion [3,33,34]. A recent postmortem study of spleens and lungs obtained from patients dying of sepsis demonstrated findings highly consistent with T cell exhaustion [3,10]. These findings included severely depressed splenocyte cytokine production, decreased T cell IL-7 receptor (CD127) expression, and increased PD-1 and PD-L1 expression on T cells and macrophages, respectively. These postmortem studies also demonstrated that PD-L1 was highly expressed on tissue parenchymal cells, that is, on splenic endothelial and bronchial epithelial cells, thereby providing opportunity for PD-1 activation [3]. Guignant and colleagues documented a correlation between PD-1 expression on circulating immune cells of septic patients and decreased T cell proliferative capacity, increased nosocomial infections and mortality [35]. Zhang *et al*. reported that anti-PD-1 was increased on monocytes from septic patients and that anti-PD-1 antibody decreased T cell apoptosis and improved immune effector function [36]. A recent important study by Singh *et al*. showed that *in vitro* blockade of PD-1 improved T cell IFN-γ production and decreased apoptosis in patients with active infections due to *M. tuberculosis*[37]. A second major finding of these investigators was that when patients with active tuberculosis were treated with effective medication to eradicate *M. tuberculosis*, the number of PD-1-expressing T cells decreased and inversely correlated with IFN-γ T-cell response against *M. tuberculosis*. We believe that this work has major implications for the broader field of sepsis because of the similarities of active tuberculosis with protracted sepsis.

In addition to data that T cell exhaustion exists in patients with chronic viral infections and sepsis, there is evidence from animal studies that treatment with anti-PD-1 and anti-PD-L1 antibodies can reverse T cell dysfunction, increase pathogen clearance and improve survival. Four different investigative teams reported that blockade of the PD-1 pathway prevents apoptotic cell death, restores host immunity and decreases mortality in clinically-relevant models of bacterial and fungal sepsis [16-20]. Given that T cell exhaustion is postulated to occur after chronic antigen exposure, it is somewhat surprising that anti-PD-1 and anti-PD-L1 antibodies were effective in particular animal models of sepsis even though the antibodies were administered relatively quickly after sepsis began, that is, often within the first 24 to 48 h after sepsis onset. These findings suggest either that other unidentified PD-1 mediated immunosuppressive mechanisms arise quickly after sepsis or that the term “exhaustion” should be more narrowly restricted. Some investigators prefer the term immune “reprioritization” rather than immune “exhaustion” in this setting. Despite this controversy, the present results showing that anti-PD-1 and anti-PD-L1 antibodies restore cytokine production and prevent apoptosis in immune cells from patients with sepsis are highly consistent with these animal studies and underscore their potential efficacy in clinical sepsis. The effect of anti-PD-1 and anti-PD-L1 to improve IFN-γ production by T cells may be particularly beneficial in sepsis given its ability to improve monocyte function, which is impaired in sepsis [4,38,39]. A clinical trial of IFN- γ in sepsis is currently underway and is being targeted to those patients whose circulating monocytes have low HLA-DR expression, (see clinicaltrials.gov Trial number NCT01649921).

An important factor in the potential clinical utility of anti-PD-1 or anti-PD-L1 antibodies in sepsis is identifying which patients would be optimal candidates for blocking therapy. Anti-PD-1 antibody has been highly successful in a subset of patients with various types of malignancies [14,15]. In general, those patients whose tumors expressed PD-L1 on immunohistochemical analysis have responded to therapy with anti-PD-1 antibody. As PD-1 and PD-L1 can also be early activation markers, it is inadvisable to use these markers alone to diagnose an immunosuppressive state. Currently, patients with sepsis whose monocytes have decreased HLA-DR expression and/or patients whose LPS-stimulated whole blood response shows decreased TNF-α production are considered good candidates for immuno-stimulatory therapy [4]. Increased CD8 T cell PD-1 expression in conjunction with these two criteria might identify patients who are good candidates for anti-PD-1 antibody in sepsis. Recent studies, as well as work from our own investigations, have shown that patients with sepsis who have a persistently low absolute lymphocyte count have a greatly increased risk of dying of sepsis [4, 6 and unpublished data]. We postulate that these patients would be ideal candidates for anti-PD-1 antibody. The positive correlation between PD-1 expression on CD4 T cells and apoptosis (Figure 9C), as well as the potent anti-apoptotic effect of anti-PD-1 suggests that anti-PD-1 would be highly advantageous in this setting by acting to increase lymphocyte numbers and function.

It is interesting to note that critically-ill non-septic patients had increased expression of PD-1 on CD4 and CD8 T cells (see Figure 1) compared to results in healthy volunteers (unpublished data). In addition to sepsis, trauma and major surgery are known to lead to a state of immunosuppression [5,28] and it is possible that PD-1:PD-L1 may be contributing to impaired host immunity in this setting as well. Conceivably, critically-ill non-septic patients who have persistent elevation of lymphocyte PD-1 expression and who are at high risk of infection might be candidates for therapy with anti-PD-1 antibodies to boost their immunity and prevent or ameliorate these infections.

A surprising finding was the potent effect of anti-PD-1 and anti-PD-L1 antibodies to increase production of IFN-γ in NKT cells from septic patients (Figure 6). Sepsis severely suppressed IFN-γ by NKT cells (Figure 5) and both anti-PD-1 and anti-PD-L1 increased the percent of IFN-γ positive T cells by approximately 50% in septic patients (Figure 6). Although the data on the role of NKT cells in sepsis are conflicting, recent studies indicate that NKT cells bridge the gap between innate and adaptive immunity and play an important role in response to particular classes of pathogens, including *Streptococcus pneumonia*, a very common cause of community acquired pneumonia [40]. NKT cells have also recently been shown to play an important role in regulating peritoneal macrophage phagocytic function in a murine sepsis model [41]. Therefore, these findings, showing a potent effect of anti-PD-1 and anti-PD-L1 in patient PBMCs, are highly relevant.

Anti-PD-1 and anti-PD-L1 antibodies have had extraordinary success in cancer trials and are considered to represent a major breakthrough in the field [42]. Anti-PD-1 antibody induced remission in approximately 20 to 25% of patients with a diversity of tumors, including malignant melanoma, renal cell cancer and non-small cell lung cancer. A remarkable feature of anti-PD-1 and anti-PD-1 therapy is the fact that some patients have durable cancer remissions that last for many months in the absence of continued therapy [43]. Cancer and sepsis share many of the same immunosuppressive mechanisms, including increased T regulatory cells, increased myeloid derived suppressor cells, and T cell exhaustion [4-8,44]. This commonality in immune pathology in cancer and sepsis could be due to the fact that both cancer and sepsis may evolve into states of chronic low grade inflammation and persistent antigen exposure. Therefore, immunotherapy that is effective in reversing immune dysfunction in cancer might have similar effects in sepsis. This finding could explain why anti-PD-1 and anti-PD-L1 are effective in these two seemingly disparate disorders. Both anti-PD-1 and anti-PD-L1 antibodies have been well tolerated in clinical trials to date [14,15,45]. Although serious autoimmune reactions can occur in patients treated with anti-PD-1 or anti-PD-L1 antibodies, these reactions are uncommon. Patients with sepsis typically may not require as prolonged a therapy with anti-PD-1/anti-PD-L1 as patients with cancer. Therefore, severe autoimmune reactions will likely be less of a problem in patients with sepsis.

## Conclusions

In conclusion, anti-PD-1 and anti-PD-L1 antibodies ameliorated key immune defects consistent with reversal of T cell exhaustion in PBMCs from septic patients. Both antibodies appeared equally effective in their capabilities. Thus, lymphocyte PD-1 expression, in conjunction with other cellular markers and clinical and laboratory findings, may contribute to identifying septic patients in which anti-PD-1 or anti-PD-L1 antibody therapy may be beneficial. Collectively, the present findings indicate that T cell exhaustion is a major etiology of immune dysfunction in sepsis and that reversal of putative T cell exhaustion using anti-PD-1 or anti-PD-L1 offers promise in the therapy of this highly lethal disorder.

## Key messages

•Sepsis induces an increase in the negative co-stimulatory molecules PD-1 and PD-L1 on patient immune effector cells.

•Blockade of the PD-1:PD-L1 pathway in septic patient peripheral blood mononuclear cells improved the ability of immune effector cells to produce key cytokines and prevented apoptotic cell death.

•Anti-PD-1 or anti-PD-L1 antibodies reverse evidence of immune cell exhaustion in sepsis and may represent a novel therapeutic approach to this life threatening disorder.

## Abbreviations

ALC: Absolute lymphocyte count; CD: Cluster of differentiation; CINS: Critically-ill non-septic; HLA-DR: Human leukocyte antigen-DR; IFN-γ: Interferon gamma; IL-2: Interleukin 2; IQR: Interquartile range; NK: Natural killer cells; NKT: Natural killer T cells; PBMCs: Peripheral blood mononuclear cells; PD-1: Programmed cell death 1; PD-L1: Programmed cell death ligand 1; TCR: T cell receptor.

## Competing interests

Dr. Hotchkiss has received research laboratory funding from MedImmune, Bristol Meyers Squibb, Pfizer, Agennix, Aurigene and the National Institutes of Health grants GM055194 and GM044118. Catherine Svabek is an Associate Scientist at MedImmune. Drs. Robbins, Ulbrandt, Suzich, Blair, Patera, and Krishnan are also employees of MedImmune.

## Authors’ contributions

KC helped design the studies, performed flow cytometry and analyzed data. CV and BS enrolled patients and entered data. CS, PR, NU, AP and JS helped analyze data. DR and SW entered and helped analyze data. JG helped design the studies and write the manuscript. RH, SK, JG, JS, PR, AP and WB helped design the studies, analyze data, and write the manuscript. All authors read and approved the final manuscript.

## Authors’ information

Drs. Subramaniam Krishnan and Richard Hotchkiss are co-senior authors.

## Supplementary Material

Additional file 1: Table S1 Septic patients. Click here for file

Additional file 2: Table S2 Critically ill non-septic patients. Click here for file

Additional file 3: Figure S1 Lymphocyte PD-1 and monocyte PD-L1 expression quantitated by flow cytometry. Peripheral blood mononuclear cells (PBMCs) from a critically-ill non-septic patient (CINS) and a septic patient were stained for programmed cell death 1 (PD-1) and programmed cell death ligand 1 (PD-L1). Lymphocytes were identified by their characteristic forward and side scatter properties (see Figure 3). Monocytes were identified by forward and side scatter properties and by CD14 immunostaining. The gray curve represents the isotype control antibody. Note the increase in the percent of lymphocytes that are PD-1 positive in septic vs CINS patients. The geo mean fluorescent intensity (MFI) is also slightly increased in septic vs CINS lymphocytes. There is also an increase in the percent of monocytes in septic patients that are PD-L1+ and an increase in the MFI as well. Click here for file

Additional file 4: Figure S2 Decreased monocyte HLA-DR in septic patients. Peripheral blood mononuclear cells (PBMCs) from critically-ill non-septic (CINS) and septic patients had immunostaining for the monocyte marker CD14 and for HLA-DR expression. Septic patients were followed sequentially during their septic illness, that is, days 1 to 3 (septic A), days 4 to 7 (septic B), days 8 to 12 (septic C) and days 12 to 21 (septic D). Note the decrease in monocyte HLA-DR expression in septic vs. CINS patients. Mean per group is indicated by horizontal bar and represent the comparison of septic samples with CINS for each draw. *P*-values shown are comparison of septic samples with CINS for each draw. Click here for file

Additional file 5: Figure S3 PD-1 and PD-L1 expression in sepsis as markers of immunosuppression 3A. Since programmed cell death 1 (PD-1) and programmed cell death ligand 1 (PD-L1) can also be activation markers, data were further separated into a CD8+ PD-1^high^ PD-L1^low^ subset defined as CD8+ PD-1 ≥36% and CD8+ PD-L1 ≤5% expression (n = 22 samples), and a CD8+ PD-1^low^ PD-L1^high^ subset defined as CD8+ PD-1 ≤36% and CD8+ PD-L1 ≥5% expression (n = 47 samples), based on levels above the mean CD8+ PD-1 and below the mean CD8+ PD-L1 expression for critically-ill non-septic controls. None of the critically-ill non-septic (CINS) patient samples were CD8+ PD-1^high^ PD-L1^low^. Selection of septic patient samples expressing high PD-1 and low PD-L1 on CD8+ T cells (CD8+ PD-1^high^ PD-L1^low^, shown in boxed region) revealed a significantly lower level of percent HLA-DR + CD14+ monocytes compared with the CD8+ PD-1^low^ PD-L1^high^ subset, indicative of a generally more immune suppressed state. Mean per group is indicated by the horizontal bars. 3B) Septic patients were separated into CD8+ PD-1^high^ PD-L1^low^ and CD8+ PD-1^low^ PD-L1^high^ subsets based on PD-1 and PD-L1 immunostaining as described above. Where multiple samples were drawn from patients over the course of their ICU stay, samples were scored as positive only once. Groups were analyzed for presence of more than two pathogens, secondary infections, type and route of infection (VAP or peritonitis). The percentage of patients positive for each parameter tested are shown for the CD8+ PD-1^high^ PD-L1^low^ and CD8+ PD-1^low^ PD-L1^high^ subsets. This data analysis revealed an increased number of secondary infections, VAP and peritonitis in septic patients with a CD8+ PD-1^high^ PD-L1^low^ phenotype (n = 14 patients) compared with a CD8+ PD-1^low^ PD-L1^high^ phenotype (n = 21 patients). VAP, ventilator associated pneumonia; G pos, Gram positive; G neg, Gram negative. Click here for file

Additional file 6: Figure S4 Sepsis induced lymphocyte apoptosis quantitated by Tunel assay. Peripheral blood mononuclear cells (PBMCs) from septic and critically-ill non-septic (CINS) patients were incubated overnight and the following morning had immunostaining for CD4 and CD8; apoptosis was quantitated by the Tunel assay. Note the increase in apoptotic (Tunel +) lymphocytes (total lymphocytes identified by forward and side scatter properties on flow cytometry), and in CD4 and CD8 T cells in septic vs. CINS patients. The maximum time point for apoptosis is during the first three days of sepsis (septic A). Mean per group is indicated by horizontal bar. *P*-values shown are comparison of septic samples with CINS for each draw. Click here for file

Additional file 7: Figure S5 Lack of correlation of PD-1 expression and absolute lymphocyte count in sepsis. Activation of the programmed cell death 1 (PD-1): programmed cell death ligand 1 (PD-L1) signaling pathway induces apoptosis in lymphocytes and thus may lead to a loss in absolute lymphocyte count (ALC). Therefore, we examined if there was a correlation between PD-1 expression on CD4 or CD8 T cells and the ALC. Freshly isolated Peripheral blood mononuclear cells (PBMCs) were obtained from septic patients throughout their septic illness and underwent immunostaining for CD4, CD8 and PD-1 as described. Note that there was no correlation between PD-1 expression on CD4 or CD8 T cells and the ALC. Click here for file

Additional file 8: Figure S6 Lack of correlation of PD-1 expression and total CD4 or CD8 T cell count. Given that programmed cell death 1 (PD-1) can induce apoptosis of lymphocytes, we examined the correlation between PD-1 expression on CD4 or CD8 T cells and the absolute numbers of CD4 or CD8 T cells. Freshly isolated peripheral blood mononuclear cells (PBMCs) were obtained from septic patients throughout their septic illness and underwent immunostaining for CD4, CD8 and PD-1 as described. Although there was a trend toward a correlation between PD-1 expression on CD4 T cells and the total number of circulating CD4 T cells, that is, the total number of circulating CD4 T cells was lowest in patients whose CD4 T cells expressed PD-1, this correlation did not reach statistical significance, (*P* = 0.12), there was no correlation between PD-1 expression on CD8 T cells and the absolute numbers of CD8 T cells. Click here for file

## References

[B1] Cohen J Opal S Calandra T Sepsis studies need new direction Lancet Infect Dis 2012 12 503 505 10.1016/S1473-3099(12)70136-6 22742624

[B2] Ward PA Immunosuppression in sepsis JAMA 2011 306 2618 2619 10.1001/jama.2011.1831 22187286

[B3] Boomer JS To K Chang KC Takasu O Osborne DF Walton AH Bricker TL Jarman SD 2nd Kreisel D Krupnick AS Srivastava A Swanson PE Green JM Hotchkiss RS Immunosuppression in patients who die of sepsis and multiple organ failure JAMA 2011 306 2594 2605 10.1001/jama.2011.1829 22187279 PMC3361243

[B4] Monneret G Venet F Pachot A Lepape A Monitoring immune dysfunctions in the septic patient: a new skin for the old ceremony Mol Med 2008 14 64 78 18026569 10.2119/2007-00102.Monneret PMC2078557

[B5] Munford RS Pugin J Normal responses to injury prevent systemic inflammation and can be immunosuppressive Am J Respir Crit Care Med 2001 163 316 321 10.1164/ajrccm.163.2.2007102 11179099

[B6] Felmet KA Hall MW Clark RS Jaffe R Carcillo JA Prolonged lymphopenia, lymphoid depletion, and hypoprolactinemia in children with nosocomial sepsis and multiple organ failure J Immunol 2005 174 3765 3772 15749917 10.4049/jimmunol.174.6.3765

[B7] Venet F Chung CS Monneret G Huang X Horner B Garber M Ayala A Regulatory T cell populations in sepsis and trauma J Leukoc Biol 2008 83 523 535 17913974 10.1189/jlb.0607371

[B8] Delano MJ Scumpia PO Weinstein JS Coco D Nagaraj S Kelly-Scumpia KM O’Malley KA Wynn JL Antonenko S Al-Quran SZ Swan R Chung CS Atkinson MA Ramphal R Gabrilovich DI Reeves WH Ayala A Phillips J Laface D Heyworth PG Clare-Salzler M Moldawer LL MyD88-dependent expansion of an immature GR-1(+)CD11b(+) population induces T cell suppression and Th2 polarization in sepsis J Exp Med 2007 204 1463 1474 10.1084/jem.20062602 17548519 PMC2118626

[B9] Day CL Kaufmann DE Kiepiela P Brown JA Moodley ES Reddy S Mackey EW Miller JD Leslie AJ DePierres C Mncube Z Duraiswamy J Zhu B Eichbaum Q Altfeld M Wherry EJ Coovadia HM Goulder PJ Klenerman P Ahmed R Freeman GJ Walker BD PD-1 expression on HIV-specific T cells is associated with T-cell exhaustion and disease progression Nature 2006 443 350 354 10.1038/nature05115 16921384

[B10] Wherry EJ T cell exhaustion Nat Immunol 2011 12 492 499 21739672 10.1038/ni.2035

[B11] Sharpe AH Wherry EJ Ahmed R Freeman GJ The function of programmed cell death 1 and its ligands in regulating autoimmunity and infection Nat Immunol 2007 8 239 245 10.1038/ni1443 17304234

[B12] Keir ME Butte MJ Freeman GJ Sharpe AH PD-1 and its ligands in tolerance and immunity Annu Rev Immunol 2008 26 677 704 10.1146/annurev.immunol.26.021607.090331 18173375 PMC10637733

[B13] Nishimura H Okazaki T Tanaka Y Nakatani K Hara M Matsumori A Sasayama S Mizoguchi A Hiai H Minato N Honjo T Autoimmune dilated cardiomyopathy in PD-1 receptor-deficient mice Science 2001 291 319 322 10.1126/science.291.5502.319 11209085

[B14] Topalian SL Hodi FS Brahmer JR Gettinger SN Smith DC McDermott DF Powderly JD Carvajal RD Sosman JA Atkins MB Leming PD Spigel DR Antonia SJ Horn L Drake CG Pardoll DM Chen L Sharfman WH Anders RA Taube JM McMiller TL Xu H Korman AJ Jure-Kunkel M Agrawal S McDonald D Kollia GD Gupta A Wigginton JM Sznol M Safety, activity, and immune correlates of anti-PD-1 antibody in cancer N Engl J Med 2012 366 2443 2454 10.1056/NEJMoa1200690 22658127 PMC3544539

[B15] Brahmer JR Tykodi SS Chow LQ Hwu WJ Topalian SL Hwu P Drake CG Camacho LH Kauh J Odunsi K Pitot HC Hamid O Bhatia S Martins R Eaton K Chen S Salay TM Alaparthy S Grosso JF Korman AJ Parker SM Agrawal S Goldberg SM Pardoll DM Gupta A Wigginton JM Safety and activity of anti-PD-L1 antibody in patients with advanced cancer N Engl J Med 2012 366 2455 2465 10.1056/NEJMoa1200694 22658128 PMC3563263

[B16] Lazar-Molnar E Gacser A Freeman GJ Almo SC Nathenson SG Nosanchuk JD The PD-1/PD-L costimulatory pathway critically affects host resistance to the pathogenic fungus Histoplasma capsulatum Proc Natl Acad Sci U S A 2008 105 2658 2663 10.1073/pnas.0711918105 18268348 PMC2268192

[B17] Huang X Venet F Wang YL Lepape A Yuan Z Chen Y Swan R Kherouf H Monneret G Chung CS Ayala A PD-1 expression by macrophages plays a pathologic role in altering microbial clearance and the innate inflammatory response to sepsis Proc Natl Acad Sci U S A 2009 106 6303 6308 10.1073/pnas.0809422106 19332785 PMC2669369

[B18] Brahmamdam P Inoue S Unsinger J Chang KC McDunn JE Hotchkiss RS Delayed administration of anti-PD-1 antibody reverses immune dysfunction and improves survival during sepsis J Leukoc Biol 2010 88 233 240 10.1189/jlb.0110037 20483923 PMC6607999

[B19] Zhang Y Zhou Y Lou J Li J Bo L Zhu K Wan X Deng X Cai Z PD-L1 blockade improves survival in experimental sepsis by inhibiting lymphocyte apoptosis and reversing monocyte dysfunction Crit Care 2010 14 R220 10.1186/cc9354 21118528 PMC3220038

[B20] Chang KC Burnham CA Compton SM Rasche DP Mazuski R Smcdonough J Unsinger J Korman AJ Green JM Hotchkiss RS Blockade of the negative co-stimulatory molecules PD-1 and CTLA-4 improves survival in primary and secondary fungal sepsis Crit Care 2013 17 R85 10.1186/cc12711 23663657 PMC3706819

[B21] Levy MM Fink MP Marshall JC Abraham E Angus D Cook D Cohen J Opal SM Vincent JL Ramsay G SCCM/ESICM/ACCP/ATS/SIS 2001 SCCM/ESICM/ACCP/ATS/SIS International Sepsis Definitions Conference Crit Care Med 2003 31 1250 1256 10.1097/01.CCM.0000050454.01978.3B 12682500

[B22] Unsinger J McGlynn M Kasten KR Hoekzema AS Watanabe E Muenzer JT McDonough JS Tschoep J Ferguson TA McDunn JE Morre M Hildeman DA Caldwell CC Hotchkiss RS IL-7 promotes T cell viability, trafficking, and functionality and improves survival in sepsis J Immunol 2010 184 3768 3779 10.4049/jimmunol.0903151 20200277 PMC2914630

[B23] Unsinger J Burnham CA McDonough J Morre M Prakash PS Caldwell CC Dunne WM Jr Hotchkiss RS Interleukin-7 ameliorates immune dysfunction and improves survival in a 2-hit model of fungal sepsis J Infect Dis 2012 206 606 616 10.1093/infdis/jis383 22693226 PMC3491749

[B24] Hotchkiss RS Swanson PE Freeman BD Tinsley KW Cobb JP Matuschak GM Buchman TG Karl IE Apoptotic cell death in patients with sepsis, shock, and multiple organ dysfunction Crit Care Med 1999 27 1230 1251 10.1097/00003246-199907000-00002 10446814

[B25] Venet F Foray AP Villars-Mechin A Malcus C Poitevin-Later F Lepape A Monneret G IL-7 restores lymphocyte functions in septic patients J Immunol 2012 189 5073 5081 10.4049/jimmunol.1202062 23053510

[B26] Kasten KR Prakash PS Unsinger J Goetzman HS England LG Cave CM Seitz AP Mazuski CN Zhou TT Morre M Hotchkiss RS Hildeman DA Caldwell CC Interleukin-7 (IL-7) treatment accelerates neutrophil recruitment through gamma delta T-cell IL-17 production in a murine model of sepsis Infect Immun 2010 78 4714 4722 10.1128/IAI.00456-10 20823197 PMC2976361

[B27] Hotchkiss RS Swanson PE Knudson CM Chang KC Cobb JP Osborne DF Zollner KM Buchman TG Korsmeyer SJ Karl IE Overexpression of Bcl-2 in transgenic mice decreases apoptosis and improves survival in sepsis J Immunol 1999 162 4148 4156 10201940

[B28] Hotchkiss RS Monneret G Payen D Immunosuppression in sepsis: a novel understanding of the disorder and a new therapeutic approach Lancet Infect Dis 2013 13 260 268 10.1016/S1473-3099(13)70001-X 23427891 PMC3798159

[B29] Christaki E Anyfanti P Opal SM Immunomodulatory therapy for sepsis: an update Expert Rev Anti Infect Ther 2011 9 1013 1033 10.1586/eri.11.122 22029521

[B30] Hotchkiss RS Opal S Immunotherapy for sepsis–a new approach against an ancient foe N Engl J Med 2010 363 87 89 10.1056/NEJMcibr1004371 20592301 PMC4136660

[B31] Hotchkiss RS Karl IE The pathophysiology and treatment of sepsis N Engl J Med 2003 348 138 150 10.1056/NEJMra021333 12519925

[B32] Hall MW Knatz NL Vetterly C Tomarello S Wewers MD Volk HD Carcillo JA Immunoparalysis and nosocomial infection in children with multiple organ dysfunction syndrome Intensive Care Med 2011 37 525 532 10.1007/s00134-010-2088-x 21153402 PMC5224706

[B33] Otto GP Sossdorf M Claus RA Rodel J Menge K Reinhart K Bauer M Riedemann NC The late phase of sepsis is characterized by an increased microbiological burden and death rate Crit Care 2011 15 R183 10.1186/cc10332 21798063 PMC3387626

[B34] Torgersen C Moser P Luckner G Mayr V Jochberger S Hasibeder WR Dunser MW Macroscopic postmortem findings in 235 surgical intensive care patients with sepsis Anesth Analg 2009 108 1841 1847 10.1213/ane.0b013e318195e11d 19448210

[B35] Guignant C Lepape A Huang X Kherouf H Denis L Poitevin F Malcus C Cheron A Allaouchiche B Gueyffier F Ayala A Monneret G Venet F Programmed death-1 levels correlate with increased mortality, nosocomial infection and immune dysfunctions in septic shock patients Crit Care 2011 15 R99 10.1186/cc10112 21418617 PMC3219369

[B36] Zhang Y Li J Zhou Y Bo L Zhu J Zhu K Wan X Cai Z Deng X Upregulation of programmed death-1 on T cells and programmed death ligand-1 on monocytes in septic shock patients Crit Care 2011 15 R70 10.1186/cc10059 21349174 PMC3222003

[B37] Singh A Mohan A Dey AB Mitra DK Inhibiting the programmed death 1 pathway rescues *Mycobacterium tuberculosis*-specific interferon γ-producing T cells from apoptosis in patients with pulmonary tuberculosis J Infectious Dis 2013 208 603 615 10.1093/infdis/jit206 23661793

[B38] Docke WD Randow F Syrbe U Krausch D Asadullah K Reinke P Volk HD Kox W Monocyte deactivation in septic patients: restoration by IFN-gamma treatment Nat Med 1997 3 678 681 10.1038/nm0697-678 9176497

[B39] Munoz C Carlet J Fitting C Misset B Bleriot JP Cavaillon JM Dysregulation of *in vitro* cytokine production by monocytes during sepsis J Clin Invest 1991 88 1747 1754 10.1172/JCI115493 1939659 PMC295719

[B40] Kinjo Y Illarionov P Vela JL Pei B Girardi E Li X Li Y Imamura M Kaneko Y Okawara A Miyazaki Y Gómez-Velasco A Rogers P Dahesh S Uchiyama S Khurana A Kawahara K Yesilkaya H Andrew PW Wong CH Kawakami K Nizet V Besra GS Tsuji M Zajonc DM Kronenberg M Invariant natural killer T cells recognize glycolipids from pathogenic Gram-positive bacteria Nat Immunol 2011 12 966 974 10.1038/ni.2096 21892173 PMC3178673

[B41] Hefferman DS Monaghan SF Thakkar RK Tran ML Chung CS Gregory SH Cioffi WG Ayala A Inflammatory mechanisms in sepsis: elevated invariant natural killer T-cell numbers in mouse and their modulatory effect on macrophage function Shock 2013 40 122 128 10.1097/SHK.0b013e31829ca519 23807244 PMC4132843

[B42] Pollack A Promising new cancer drugs empower the body’s own defense system The New York Times (Health section) 2013 [http://www.nytimes.com/2013/06/04/health/promising-new-cancer-drugs-empower-the-bodys-own-defense-system.html?pagewanted=1&_r=0]

[B43] Lipson EJ Sharfman WH Drake CG Wollner I Taube JM Anders RA Xu H Yao S Pons A Chen L Pardoll DM Brahmer JR Topalian SL Durable cancer regression off-treatment and effective reinduction therapy with an anti-PD-1 antibody Clin Cancer Res 2013 19 462 468 10.1158/1078-0432.CCR-12-2625 23169436 PMC3548952

[B44] Cheever MA Twelve immunotherapy drugs that could cure cancers Immunol Rev 2008 222 357 368 10.1111/j.1600-065X.2008.00604.x 18364014

[B45] Hamid O Robert C Hodi FS Hwu WJ Kefford R Wolchok JD Hersey P Joseph RW Weber JS Dronca R Gangadhar TC Patnaik A Zarour H Joshua AM Gergich K Elassaiss-Schaap J Algazi A Mateus C Boasberg P Tumeh PC Chmielowski B Ebbinghaus SW Li XN Kang SP Ribas A Safety and tumor responses with lambrolizumab (anti-PD-1) in melanoma N Engl J Med 2013 369 134 144 10.1056/NEJMoa1305133 23724846 PMC4126516

