# Supplementary material for: Targeting the programmed cell death 1: programmed cell death ligand 1 pathway reverses T cell exhaustion in patients with sepsis
Source: Crit Care. 2014 Jan 4;18(1):R3. doi: 10.1186/cc13176 (PMC4056005; doi:10.1186/cc13176)
Supplement: Additional file 1: Table S1 — Septic patients. [file cc13176-S1.pdf]

Supplemental Table 1 -- Septic Patients

Pt #	Age	Gender	Clinical Cond.	Days in ICU	Micro	New Secondary Infections	Total WCC	Apache II	SOFA Score
1	71	Male	elevated wcc, febrile, hypotensive, pressors, tachycardic, tachypneic, mechanical ventilation	30	Tracheal aspirate- MRSA, Pseudomonas aeruginosa	CXR- right upper lobe airspace opacity consistent with pneumonia	23.9	26	9
2	71	Male	elevated wcc, febrile, tachycardic, tachypneic	15	Stool- C.diff.	Pancreatic Abscess	13.1	14	4
3	28	Male	elevated wcc, afebrile, hypotensive, pressors, tachycardic, tachypnic, mechanical ventilation.	98	BAL- Klebsiella oxytoca	bilateral lung parenchyma patchy opacities that may represent combination of pneumonia, atelectasis, infarction, or contusion.	59.4	22	13
4	25	Female	normal wcc, febrile, hypotensive, pressors, tachycardic, tachypneic, mechanical ventilation	41	Trach Aspirate- Pseudomonas aeruginosa	wound infection	5.7	13	8
5	77	Female	elevated wcc, afebrile, hypotensive, pressors, tachypneic, increased oxygen requirements	3			11.7	16	10
6	21	Female	elevated wcc, afebrile, hypotensive, pressors, tachycardic, mechanical ventilation	20	Urine- Enterococcus species		16	21	13
7	49	Male	elevated wcc, afebrile, hypotensive, pressors, tachycardic	6	Urine- Serratia marcescens		21.1	19	15
8	73	Female	elevated wcc, febrile, hypotensive, pressors, tachycardic,tachypneic, mechanical ventilation	17	Stool- C.diff.		14.7	23	10
9	55	Male	elevated wcc, febrile, hypotensive, pressors, mechanical ventilation	11		CXR- focal nodular opacity in the left midlung which in the appropriate clinical setting may represent a developing pneumonia.	24.7	21	9
10	76	Male	elevated wcc, hypotensive, pressors, tachycardic, mechanical ventilation	4		Chest CT- multifocal pneumonia at the lung bases with a small focus of cavitation in the lingula	14.6	22	10
11	51	Female	normal wcc, febrile, tachycardic, hypotensive, pressors, tachypneic, mechanical ventilation	30	BAL- Stenotrophomonas maltophilia	CXR- Pneumonia	8.2	21	9

12	61	Female	elevated wcc, afebrile, hypotensive, pressors, tachypneic, mechanical ventilation	7	Urine- Klebsiella oxytoca		15	18	9
13	60	Female	elevated wcc, febrile, hypotensive, pressors, tachycardic, tachypneic, mechanical ventilation	12	Blood- Streptococcus pneumoniae		28.3	21	10
14	64	Female	elevated wcc, hypotensive, pressors, tachycardic	16	peritoneal tap- Enterococcus faecium		20.8	18	10
15	62	Male	elevated wcc, afebrile, recovering	7	Sputum- Haemophilus influenzae, MSSA		10.6	14	5
16	>89	Male	elevated wcc, hypotensive, pressors, tachycardic	8		CT- Perforated Cecum	14.8	21	12
17	33	Male	normal wcc, febrile, hypotensive, pressors, tachycardic, mechanical ventilation	15		CHEST CT- Pneumonia	7.1	18	9
18	67	Female	elevated wcc, afebrile, extubated	4	Urine- E.coli		10	12	5
19	56	Female	elevated wcc, afebrile, hypotensive, pressors, tachycardic, tachypneic, mechanical ventilation	6	Neck Abscess- Streptococcus anginosus		13.7	16	7
20	69	Male	elevated wcc, febrile, hypotensive, pressors, tachycardic, tachypnic, mechanical ventilation	30	BAL- Klebsiella pneumoniae, Proteus mirabilis	VAP	12	17	6
21	69	Male	elevated wcc, febrile, tachycardic, tachypneic	6	BAL- Cytomegalovirus: POSITIVE, Pseudomonas aeruginosa		17.5	16	5
22	62	Female	elevated wcc, febrile, tachypneic, mechanical ventilation	15	BLD- Corynebacterium species, Haemophilus influenzae	VAP	13.5	13	4
23	76	Male	elevated wcc, afebrile, pressors weaned off	10	BLD- Candida albicans	Fungemia	10.9	15	8
24	82	Female	elevated wcc, febrile, hypotensive, pressors, tachycardic, tachypneic, mechanical ventilation	7	BAL- Moraxella catarrhalis		14.9	19	11
25	55	Male	elevated wcc, afebrile, hypotensive, pressors, tachycardic, tachypneic	10	Abscess- VRE; Paracentesis - Enterococcus faecium	Abscess	13.9	13	6
26	69	Male	elevated wcc, febrile, tachycadic, tachypneic	NA	BLD- Candida glabrata	Fungemia	10.9	9	3
27	60	Male	elevated wcc, afebrile, tachypnic	8	BLD- Candida glabrata	Fungemia	14.8	8	3
28	63	Female	elevated wcc, afebrile, tachycardic, tachypneic, mechanical ventilation, extubated	7	Abscess- Corynebacterium species		15.4	12	4

29	74	Male	elevated wcc, afebrile, hypotensive, pressors, extubated	4			11.4	11	5
30	70	Female	elevated wcc, afebrile, hypotensive, pressors, tachypneic, mechanical ventilation	22	BLD- Enterobacter cloacae, Candida albicans	Fungemia, VAP	10.5	18	10
31	83	Female	normal wcc, afebrile, pressors weaned off, tachypneic	5			8	13	3
32	48	Female	elevated wcc, afebrile, hypotensive, pressors, tachycardic, tachypneic, mechanical ventilation	13	Urine-Enterococcus species	UTI	25.1	18	10
33	33	Female	normal wcc, afebrile, tachycardic, mechanical ventilation	11	Ear Wound- Proteus mirabilis, Staphylococcus lugdunensis		9.7	14	6
34	64	Female	elevated wcc, afebrile, stable	NA	GPC		11.7	13	6
35	44	Male	elevated wcc, afebrile, tachycardic, mechanical ventilation	5			11.3	16	9
36	44	Male	elevated wcc, febrile, hypotensive, pressors, tachycardic, tachypneic, mechanical ventilation	10	BLD- MRSA; Abscess- Streptococcus agalactiae, MRSA		18.9	33	15
37	77	Female	elevated wcc, afebrile, stable, positive fungal bld cx.	NA	BLD- Candida glabrata		10.3	10	2
38	64	Female	elevated wcc, afebrile, tachycardic	NA	BLD- Candida glabrata		11.6	8	4
39	70	Female	elevated wcc, afebrile, extubated	10	Stool- VRE; Abd Fld- Candida albicans, Bacteroides fragilis group		18.6	10	3
40	64	Female	elevated wcc, febrile, hypotensive, pressors, tachycardic, mechanical ventilation	23	BLD- Enterococcus faecium	Abscess	10.4	25	15
41	62	Male	elevated wcc, febrile, tachycardic	NA	BLD- Candida albicans	line infection	12.2	7	2
42*	83	Male	elevated wcc, febrile, hypotensive, pressors, tachycardic, tachypneic	14	trach aspirate- Haemophilus influenzae	CXR- developing Pneumonia	19.9	20	9
43*	42	Male	elevated wcc, febrile, hypotensive, pressors, tachycardic, tachypneic, mechanical ventilation	16		VAP	10.8	16	4

* Admitted as non-septic but developed sepsis

BAL, bronchial alveolar lavage; **BLD**, blood; **C. diff**, Clostridia difficile; **CT**, CT scan; **CXR**, chest x-ray; **GPC**, Gram positive cocci;

MRSA, methicillin resistant Staph. aureus; **pressors**, vaso pressors; **VAP**, ventilator associated pneumonia; **WCC**, white cell count
