# Supplementary material for: Targeting the programmed cell death 1: programmed cell death ligand 1 pathway reverses T cell exhaustion in patients with sepsis
Source: Crit Care. 2014 Jan 4;18(1):R3. doi: 10.1186/cc13176 (PMC4056005; doi:10.1186/cc13176)
Supplement: Additional file 2: Table S2 — Critically ill non-septic patients. [file cc13176-S2.pdf]

Supplemental Table 2 Critically Ill Non-Septic Patients

Patient #	Age	Gender	Clinical Cond.	Days in ICU	Total WCC	Apache II	SOFA Score
1	83	Male	normal wcc, febrile, normotensive, stable	14	9.8	7	3
2	43	Male	normal wcc, febrile, tachycardic, mechanical ventilation	7	8.5	12	4
3	82	Male	normal wcc, afebrile, stable	9	9	7	3
4	51	Male	normal wcc, afebrile, mechanical ventilation	7	8.8	8	4
5	73	Female	normal wcc, afebrile, tachycardic, mechanical ventilation	7	8.7	10	4
6	81	Female	elevated wcc, afebrile, hypotensive, pressors, tachypnic, mechanical ventilation	2	10.9	17	7
7	42	Female	elevated wcc, afebrile, tachycardic, stable	2	10	5	1
8	85	Male	normal wcc, afebrile, stable	2	5.2	8	3
9	31	Male	elevated wcc, afebrile, stable	6	18.6	6	2
10	29	Male	elevated wcc, afebrile, stable	3	13.9	3	1
11	64	Female	normal wcc, afebrile, stable	3	8.5	8	3
12	42	Male	normal wcc, febrile, tachycardic	16	5.8	12	5
13	53	Female	low wcc, afebrile, stable	5	3.3	7	2
14	57	Male	normal wcc, afebrile, tachypnic, stable	3	4.5	6	1
15	39	Female	elevated wcc, afebrile, tachycardic, stable	2	12.4	2	0
