# Supplementary figures and images for: Targeting the programmed cell death 1: programmed cell death ligand 1 pathway reverses T cell exhaustion in patients with sepsis
Source: Crit Care. 2014 Jan 4;18(1):R3. doi: 10.1186/cc13176 (PMC4056005; doi:10.1186/cc13176)

# Supplemental Fig. 1

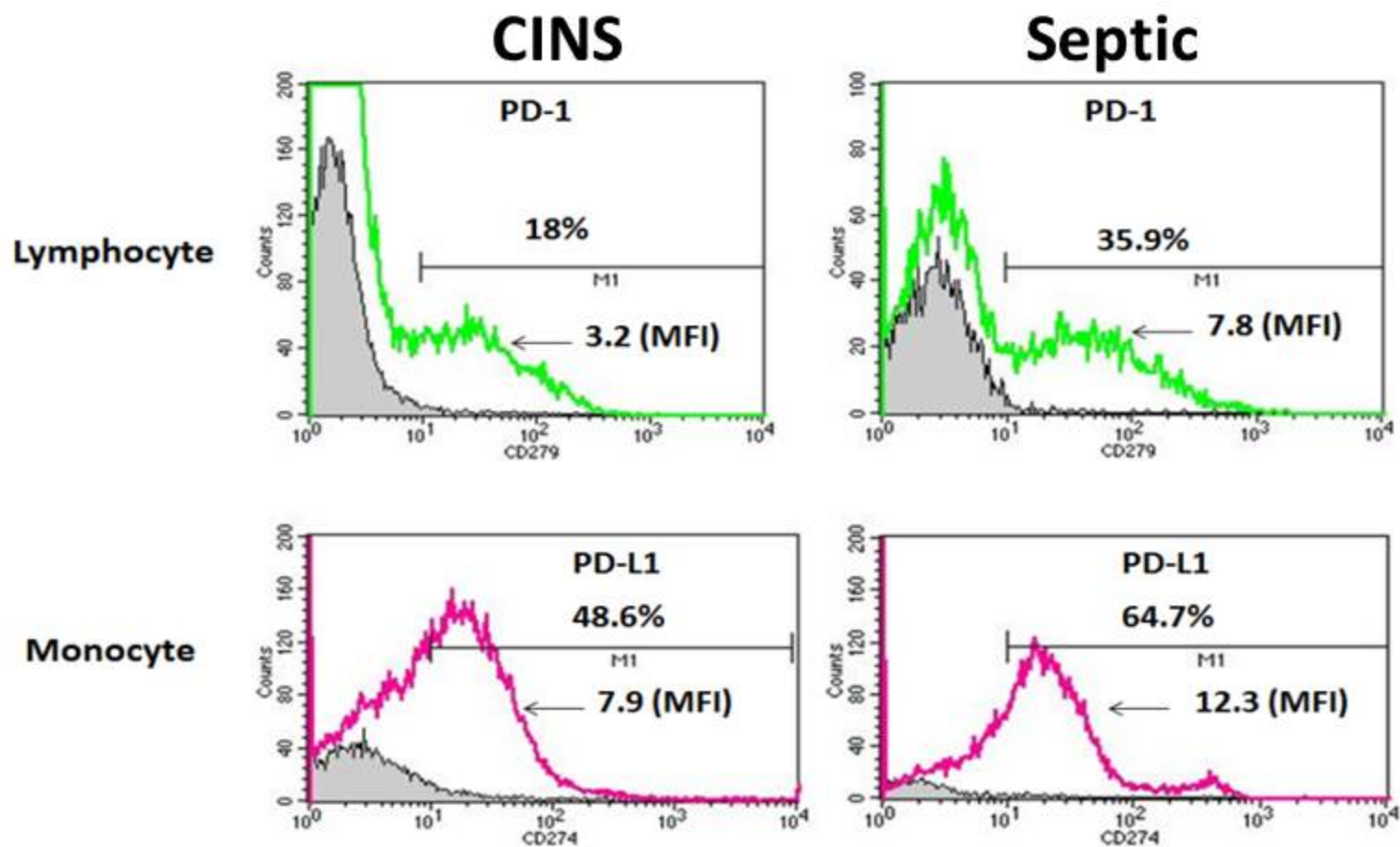

Supplement: Additional file 3: Figure S1 — Lymphocyte PD-1 and monocyte PD-L1 expression quantitated by flow cytometry. Peripheral blood mononuclear cells (PBMCs) from a critically-ill non-septic patient (CINS) and a septic patient were stained for programmed cell death 1 (PD-1) and programmed cell death ligand 1 (PD-L1). Lymphocytes were identified by their characteristic forward and side scatter properties (see Figure 3). Monocytes were identified by forward and side scatter properties and by CD14 immunostaining. The gray curve represents the isotype control antibody. Note the increase in the percent of lymphocytes that are PD-1 positive in septic vs CINS patients. The geo mean fluorescent intensity (MFI) is also slightly increased in septic vs CINS lymphocytes. There is also an increase in the percent of monocytes in septic patients that are PD-L1+ and an increase in the MFI as well. [file cc13176-S3.pdf]

## Supplemental Fig. 2

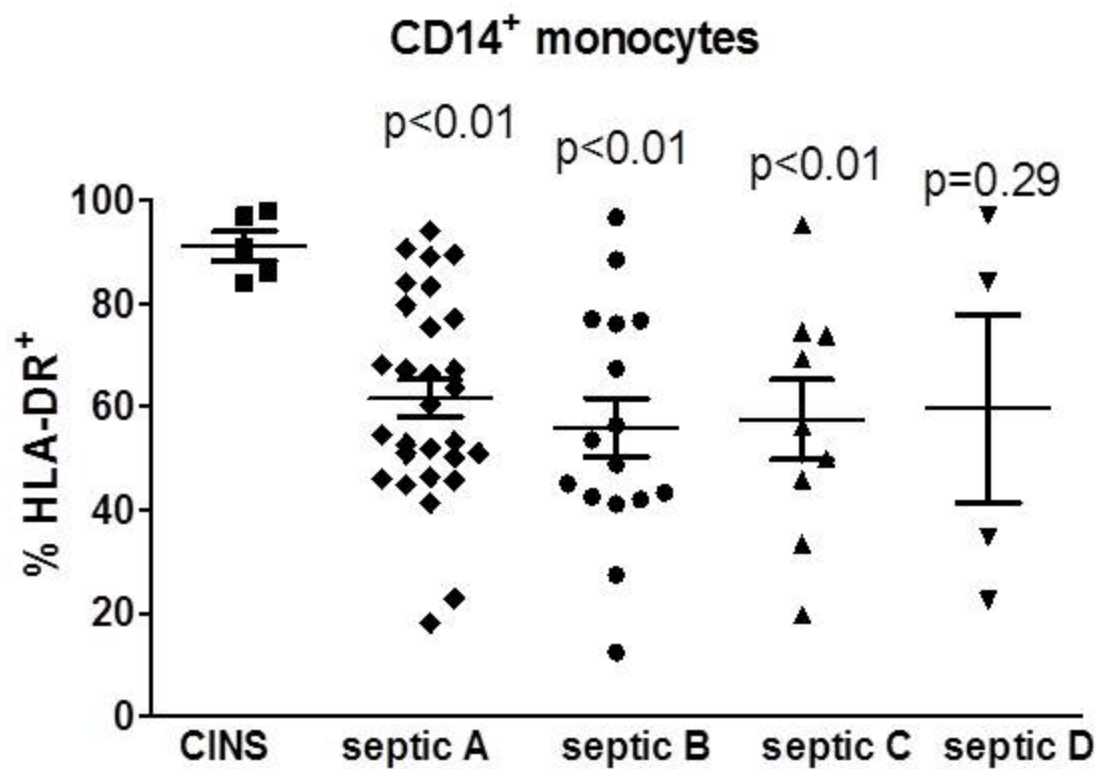

Supplement: Additional file 4: Figure S2 — Decreased monocyte HLA-DR in septic patients. Peripheral blood mononuclear cells (PBMCs) from critically-ill non-septic (CINS) and septic patients had immunostaining for the monocyte marker CD14 and for HLA-DR expression. Septic patients were followed sequentially during their septic illness, that is, days 1 to 3 (septic A), days 4 to 7 (septic B), days 8 to 12 (septic C) and days 12 to 21 (septic D). Note the decrease in monocyte HLA-DR expression in septic vs. CINS patients. Mean per group is indicated by horizontal bar and represent the comparison of septic samples with CINS for each draw. P-values shown are comparison of septic samples with CINS for each draw. [file cc13176-S4.pdf]

Supplemental Fig. 3

A

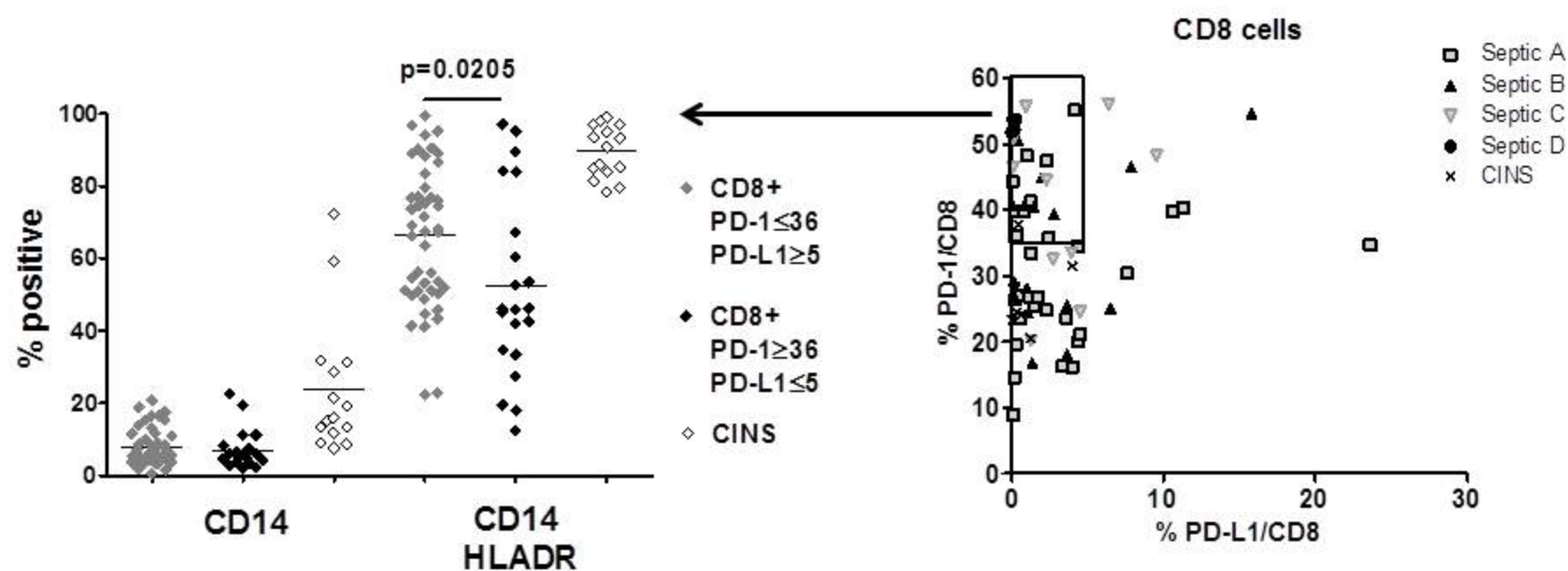

B

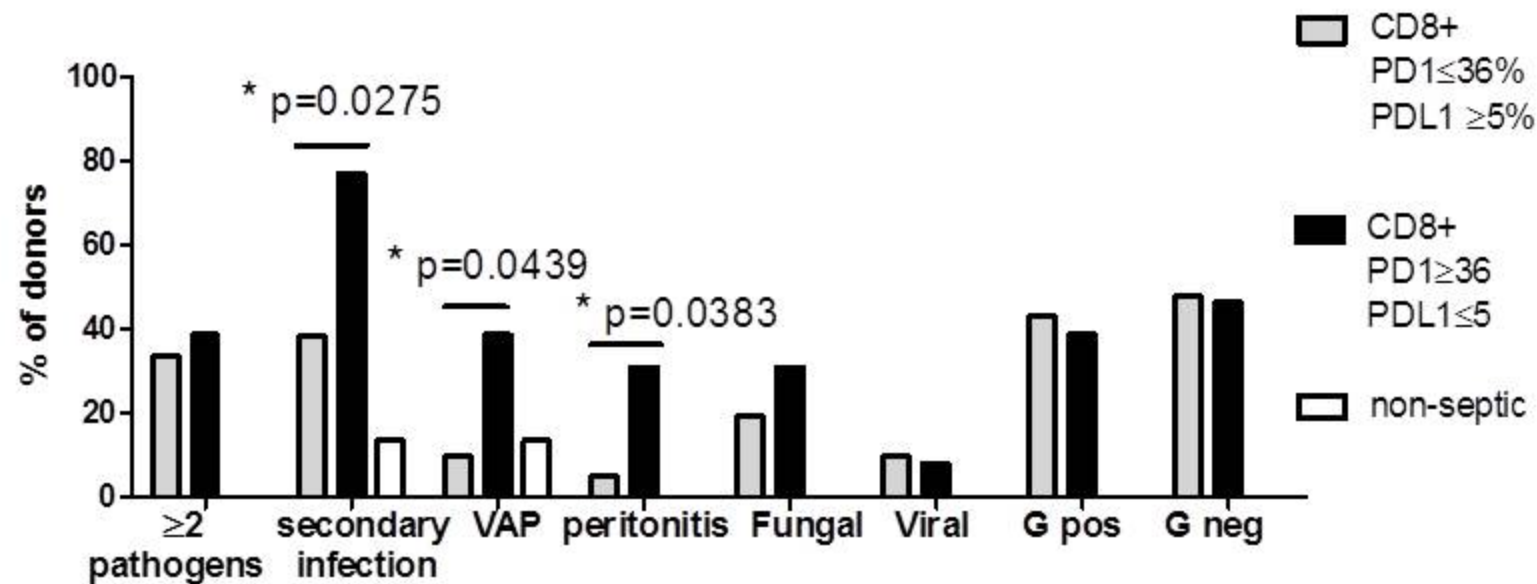

Supplement: Additional file 5: Figure S3 — PD-1 and PD-L1 expression in sepsis as markers of immunosuppression 3A. Since programmed cell death 1 (PD-1) and programmed cell death ligand 1 (PD-L1) can also be activation markers, data were further separated into a CD8+ PD-1high PD-L1low subset defined as CD8+ PD-1 ≥36% and CD8+ PD-L1 ≤5% expression (n = 22 samples), and a CD8+ PD-1low PD-L1high subset defined as CD8+ PD-1 ≤36% and CD8+ PD-L1 ≥5% expression (n = 47 samples), based on levels above the mean CD8+ PD-1 and below the mean CD8+ PD-L1 expression for critically-ill non-septic controls. None of the critically-ill non-septic (CINS) patient samples were CD8+ PD-1high PD-L1low. Selection of septic patient samples expressing high PD-1 and low PD-L1 on CD8+ T cells (CD8+ PD-1high PD-L1low, shown in boxed region) revealed a significantly lower level of percent HLA-DR + CD14+ monocytes compared with the CD8+ PD-1low PD-L1high subset, indicative of a generally more immune suppressed state. Mean per group is indicated by the horizontal bars. 3B) Septic patients were separated into CD8+ PD-1high PD-L1low and CD8+ PD-1low PD-L1high subsets based on PD-1 and PD-L1 immunostaining as described above. Where multiple samples were drawn from patients over the course of their ICU stay, samples were scored as positive only once. Groups were analyzed for presence of more than two pathogens, secondary infections, type and route of infection (VAP or peritonitis). The percentage of patients positive for each parameter tested are shown for the CD8+ PD-1high PD-L1low and CD8+ PD-1low PD-L1high subsets. This data analysis revealed an increased number of secondary infections, VAP and peritonitis in septic patients with a CD8+ PD-1high PD-L1low phenotype (n = 14 patients) compared with a CD8+ PD-1low PD-L1high phenotype (n = 21 patients). VAP, ventilator associated pneumonia; G pos, Gram positive; G neg, Gram negative. [file cc13176-S5.pdf]

Supplemental Fig. 4

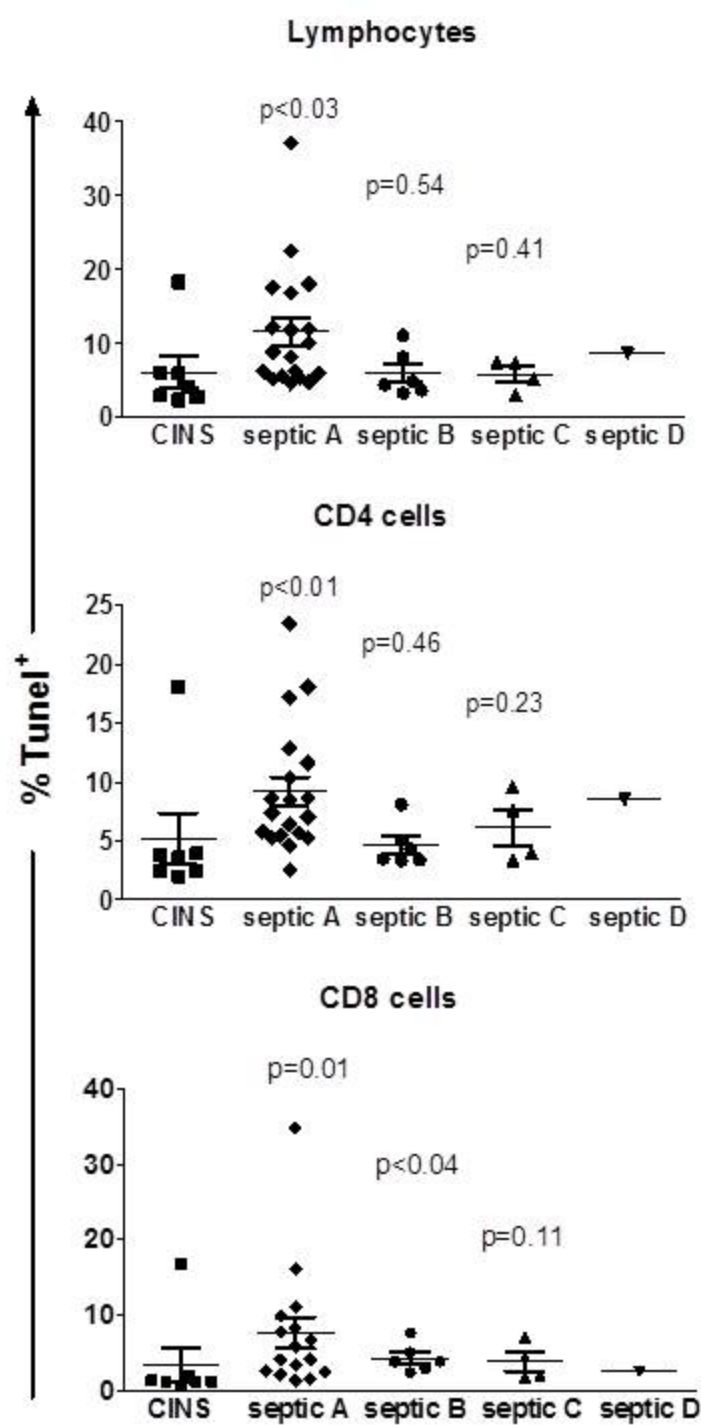

Supplement: Additional file 6: Figure S4 — Sepsis induced lymphocyte apoptosis quantitated by Tunel assay. Peripheral blood mononuclear cells (PBMCs) from septic and critically-ill non-septic (CINS) patients were incubated overnight and the following morning had immunostaining for CD4 and CD8; apoptosis was quantitated by the Tunel assay. Note the increase in apoptotic (Tunel +) lymphocytes (total lymphocytes identified by forward and side scatter properties on flow cytometry), and in CD4 and CD8 T cells in septic vs. CINS patients. The maximum time point for apoptosis is during the first three days of sepsis (septic A). Mean per group is indicated by horizontal bar. P-values shown are comparison of septic samples with CINS for each draw. [file cc13176-S6.pdf]

Supplemental Fig. 5

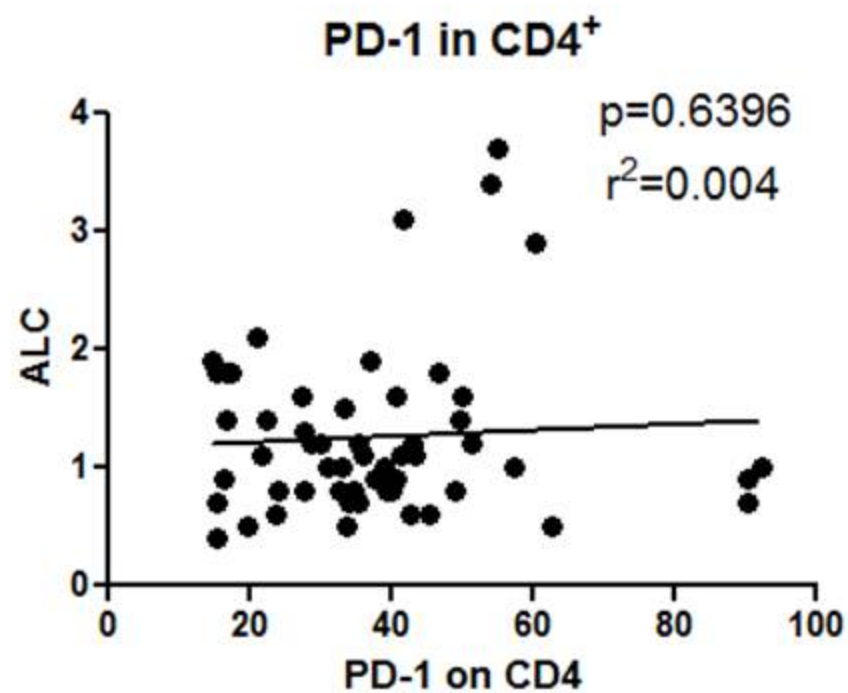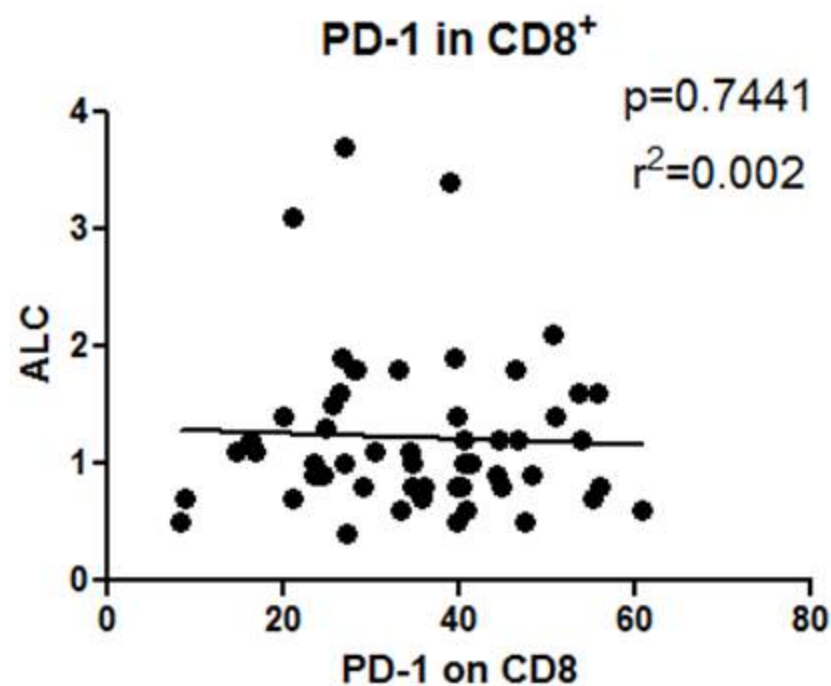

Supplement: Additional file 7: Figure S5 — Lack of correlation of PD-1 expression and absolute lymphocyte count in sepsis. Activation of the programmed cell death 1 (PD-1): programmed cell death ligand 1 (PD-L1) signaling pathway induces apoptosis in lymphocytes and thus may lead to a loss in absolute lymphocyte count (ALC). Therefore, we examined if there was a correlation between PD-1 expression on CD4 or CD8 T cells and the ALC. Freshly isolated Peripheral blood mononuclear cells (PBMCs) were obtained from septic patients throughout their septic illness and underwent immunostaining for CD4, CD8 and PD-1 as described. Note that there was no correlation between PD-1 expression on CD4 or CD8 T cells and the ALC. [file cc13176-S7.pdf]

## Supplemental Fig. 6.

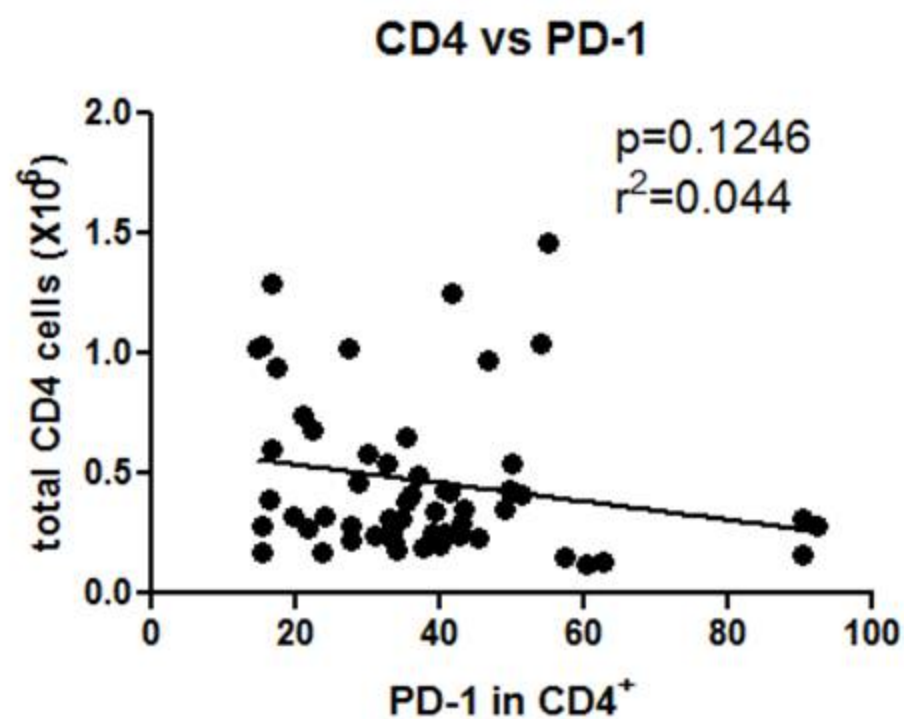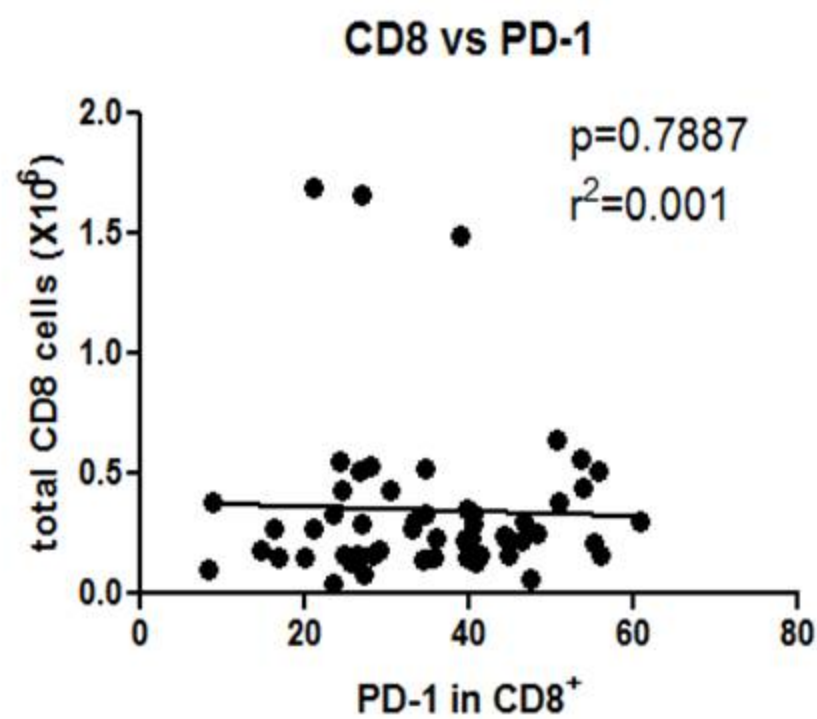

Supplement: Additional file 8: Figure S6 — Lack of correlation of PD-1 expression and total CD4 or CD8 T cell count. Given that programmed cell death 1 (PD-1) can induce apoptosis of lymphocytes, we examined the correlation between PD-1 expression on CD4 or CD8 T cells and the absolute numbers of CD4 or CD8 T cells. Freshly isolated peripheral blood mononuclear cells (PBMCs) were obtained from septic patients throughout their septic illness and underwent immunostaining for CD4, CD8 and PD-1 as described. Although there was a trend toward a correlation between PD-1 expression on CD4 T cells and the total number of circulating CD4 T cells, that is, the total number of circulating CD4 T cells was lowest in patients whose CD4 T cells expressed PD-1, this correlation did not reach statistical significance, (P = 0.12), there was no correlation between PD-1 expression on CD8 T cells and the absolute numbers of CD8 T cells. [file cc13176-S8.pdf]
